# Enhancing *π*-Delocalization and Suppressing Traps via Doping in Electron Transport Materials for Efficient Semitransparent Organic Photovoltaics

**DOI:** 10.1007/s40820-026-02083-1

**Published:** 2026-02-09

**Authors:** Yating Mo, Jiayu Wang, Hanjiao Chen, Yufei Gong, Jianglong Zhou, Junhao Lu, Cenqi Yan, Lei Meng, Liang-Wen Feng, Yongfang Li, Pei Cheng

**Affiliations:** 1 College of Polymer Science and Engineering, State Key Laboratory of Advanced Polymer Materials, Sichuan University, 610065 Chengdu, People’s Republic of China; 2 Analytic & Testing Center, Sichuan University, 610065 Chengdu, People’s Republic of China; 3 Beijing National Laboratory for Molecular Sciences, CAS Key Laboratory of Organic Solids, Institute of Chemistry, Chinese Academy of Sciences, 100190 Beijing, People’s Republic of China; 4 Key Laboratory of Green Chemistry & Technology, Ministry of Education, College of Chemistry, Sichuan University, 610065 Chengdu, People’s Republic of China

**Keywords:** Semitransparent organic photovoltaics, Interfacial engineering, *π*-delocalization, Surface traps, Electrical loss

## Abstract

The ultrathin metal electrode in semitransparent organic photovoltaics (STOPVs) usually suffers from limited charge collection capability and conductivity and thus hinders the power conversion efficiency (PCE). Herein, a new strategy of enhancing the *π*-delocalization of electron transport layer (ETL) via lithium bis(trifluoromethanesulfonyl)imide doping is developed. The enhanced *π*-delocalization in ETL benefits sizeable intermolecular *π*–*π* overlap, prone to harvesting electrons and thereby improving charge collection range. Doping also improves the conductivity of both ETL and ultrathin silver electrode. Furthermore, the trap densities in ETL and STOPV devices are reduced after doping, contributing to suppressed recombination and higher PCE. Consequently, ETL doping maintains an average visible transmittance of ~ 30% while promotes the PCE of STOPVs from 13.0% to 14.3% and light utilization efficiency from 3.74% to 4.15%, which is among the highest values of optical structure-free STOPVs. This work provides a new insight of π-delocalization manipulation in ETL for efficient STOPVs.

**Supplementary Information:**

The online version contains supplementary material available at 10.1007/s40820-026-02083-1.

## Highlights

- High-performance semitransparent organic photovoltaics (STOPVs) with decreased electrical loss were fabricated via introducing lithium bis(trifluoromethanesulfonyl)imide (LiTFSI) to electron transport layer (ETL).
- LiTFSI interacts with ETL material PDINN and enhanced π-delocalization in PDINN, which is beneficial to conductivity and thereby electron collection range.
- LiTFSI-doped PDINN-based STOPVs show an improved power conversion efficiency of 14.3%, average visible transmittance of 29.0%, and light utilization energy of 4.15%, which is among the highest values of optical structure-free STOPVs.

**Supplementary Information:**

The online version contains supplementary material available at 10.1007/s40820-026-02083-1.

## Graphical abstract

**Figure:**
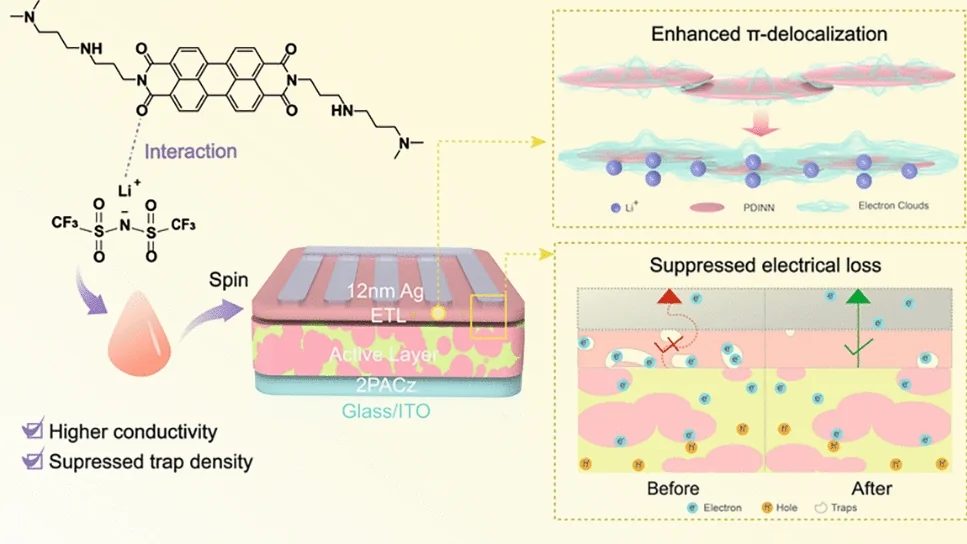

## Introduction

Semitransparent organic photovoltaics (STOPVs) leverage the discontinuous absorption properties of organic semiconductors, enabling them to possess both light transmittance and electricity generation capabilities [1–5]. Given their potential applications in a variety of settings, such as agricultural greenhouses [6, 7], smart windows [8, 9], and floating photovoltaics [10], STOPVs have gained escalating interest. The average visible transmittance (AVT) and power conversion efficiency (PCE) are two vital parameters for assessing the performance of STOPVs. The optimal PCE of opaque organic photovoltaics (OPVs) has surpassed 20% in recent years owing to molecular [11–15] and device engineering [16–20], while those of the highest-performing STOPVs with an AVT of approximately 30% still lag behind with significantly decreased short-circuit current density (*J*_SC_) [21–24]. To achieve high PCE at a given AVT, lots of effective strategies have been reported to maximize the light harvesting in near-infrared region to compensate the optical loss in visible region, such as designing new active layer materials and incorporating third components [25–32].

However, the current loss in STOPVs is not solely attributed to optical loss, and electrical loss is another significant factor responsible for performance degradation [3, 33]. The electrical loss continuously increases as the Ag electrode thickness is reduced from 100 to 10 nm, accompanied by a dramatic rise in surface trap density by three orders of magnitude [34]. Therefore, reducing surface traps and unnecessary electrical loss in STOPVs is of great importance for enhancing their photovoltaic performance. Lots of works have demonstrated that the thickness of transparent Ag electrodes is insufficient to form an efficient conductive network, although thin Ag electrodes exhibit considerable transparency and fabrication simplicity [35–37]. Near the threshold thickness, Ag typically exhibits limited charge collection range, suboptimal lateral conductivity, and increased defects, which dominate the electrical loss in STOPVs [37–39]. Interfacial engineering strategies, such as introducing seed layer [40] or covalent interaction [33], were reported to ameliorate the performance of thin Ag. To further improve the performance of STOPVs, diverse and effective approaches need to be explored. Recently, a longitudinal through-hole architecture was reported to eliminate the dependence on ultrathin metal electrode. In STOPVs with this architecture, opaque silver grid is used as top electrode and responsible for efficient charge collection, while the longitudinal holes through the device contribute to optical transparency, achieving PCEs of 6–15% with AVT of 60–10% [41].

**Scheme 1:**
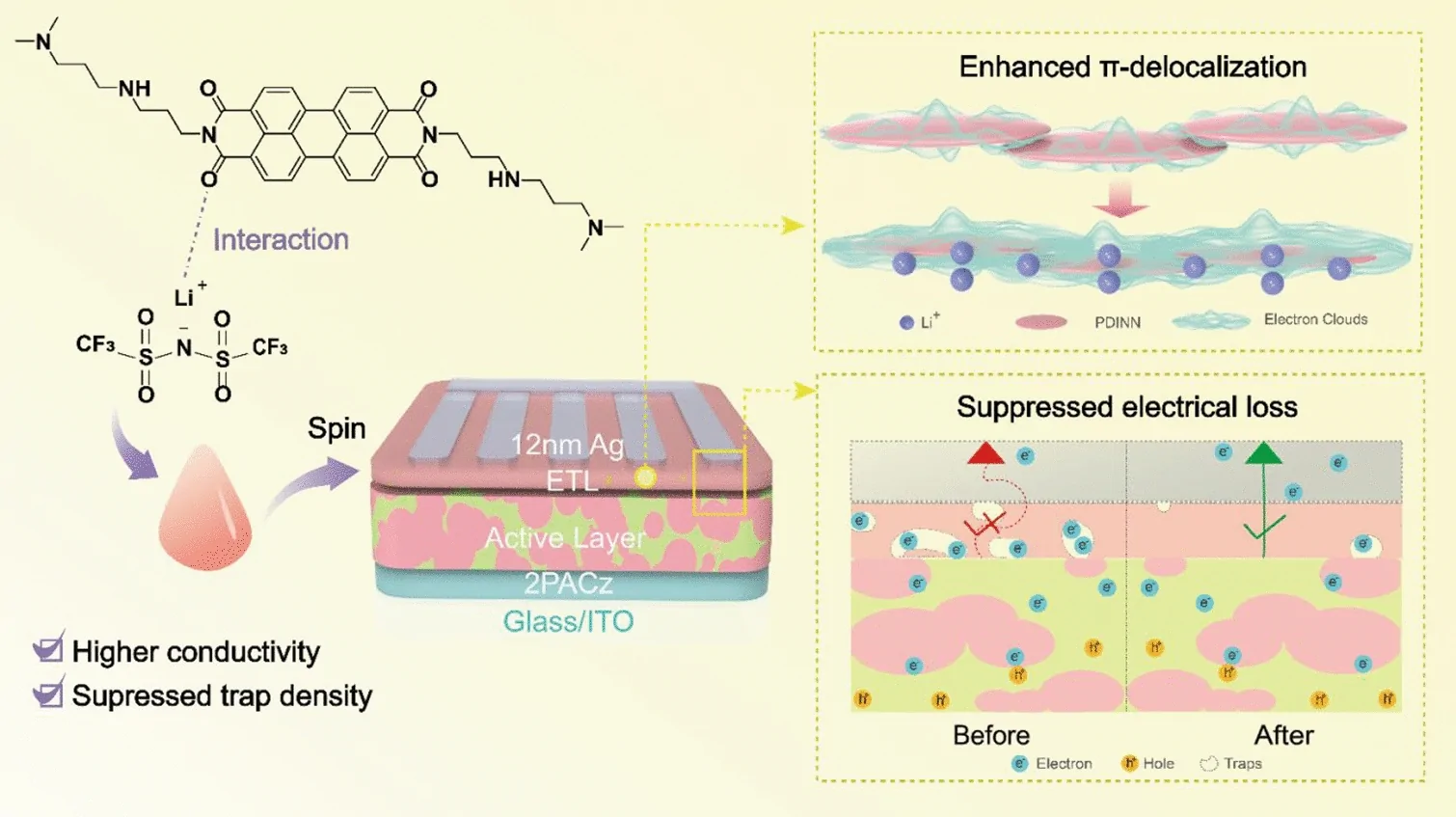
Illustration of ETL doping process and electronic distribution evolution

Herein, we proposed a new strategy of enhancing the electron collection ability and conductivity of ultrathin metal electrode via electron transport layer (ETL) doping (Scheme 1). Lithium bis(trifluoromethanesulfonyl)imide (LiTFSI), consisted of strong electron-withdrawing TFSI⁻ anions and a Li^+^ cation, is used as the dopant. LiTFSI interacts with ETL material PDINN through electrostatic interaction between Li^+^ and carbonyl group and induces a more delocalized π electron distribution in PDINN, which is beneficial to intermolecular *π*–*π* overlap and thereby electron collection range. After LiTFSI doping, the PDINN ETL exhibits improved conductivity from 9.67 × 10^−5^ to 1.71 × 10^−4^ S cm^−1^, increased doping density from 2.69 × 10^16^ to 4.01 × 10^16^ cm^−3^, and reduced trap density from 1.29 × 10^17^ to 7.26 × 10^16^ cm^−3^. Charge dynamic analysis further demonstrates that LiTFSI-doped PDINN (D-PDINN)-based STOPVs present enhanced charge extraction, suppressed charge recombination and inhibited surface traps relative to PDINN-based counterparts. As a result, compared with PDINN-based STOPVs with a PCE of 13.0%, an AVT of 28.8% and a light utilization efficiency (LUE = PCE × AVT) of 3.74%, D-PDINN-based STOPVs show an improved PCE of 14.3%, a comparable AVT of 29.0% and a higher LUE of 4.15%, which is among the highest values of optical structure-free STOPVs.

## Experimental Section

### Materials

2PACz, PM6, BTP-eC9, and PDINN were purchased from Hyper Materials. LiTFSI was purchased from Alladin Materials. PEDOT:PSS 4083 was purchased from Heraeus. PDINO and PDNIT-F3N were purchased from Solarmer.

### Device Fabrication

STOPVs were fabricated with a conventional architecture of indium tin oxide (ITO)/PEDOT:PSS/active layer/ETL/Ag (12 nm). The ITO-coated glass substrates were pre-cleaned ultrasonically in water, acetone, and ethanol in sequence for 15 min and then treated with UV-ozone for 20 min. PEDOT:PSS 4083 was spin-coated at 3000 rpm for 30 s and annealed at 150 °C for 20 min. For better device performance, 2PACz was used to replace PEDOT:PSS 4083. 2PACz (0.3 mg mL^−1^ in ethanol) was spin-coated at 3000 rpm for 30 s and annealed at 100 °C for 5 min. PM6/BTP-eC9 (1:1.5, w/w) was dissolved in CB with a total concentration of 20 mg mL^−1^, with 0.6 vol% DIO as addictive. The solution of PM6:BTP-eC9 needed to be stirred at 60 °C overnight until completely dissolved. The solution was spin-coated onto 2PACz or PEDOT:PSS 4083 modified substrate at 3200 rpm for 45 s to form active layer (80 nm). The active layer was then annealed at 100 °C for 5 min. A thin layer (~ 5 nm) of PDINN in methanol with a concentration of 1 mg mL^−1^ was spin-coated on the top of the active layer at 3000 rpm for 30 s. For PDINO-based STOPVs, PDINO was spin-coated at 3000 rpm for 30 s with a concentration of 1 mg mL^−1^ in methanol. For PNDIT-F3N-based STOPVs, ETL was spin-coated at 1500 rpm for 30s with a concentration of 1.5 mg mL^−1^ in methanol (0.5 vol% Acetic acid). Finally, 12 nm Ag was evaporated sequentially under high vacuum.

### Device Characterization and Analysis

*J–V* characteristics were measured in the forward direction from − 0.5 to 1.5 V using computer-controlled Keysight B2901A Source Meter under the illumination of an Enlitech solar simulator (SS-X50, AAA grade) coupled with AM 1.5G solar spectrum filter in a nitrogen glove box at room temperature. The light intensity was calibrated to 100 mW cm^−2^ using a standard silicon reference cell (SRC2020). The devices were tested under a mask with an area of 2.56 mm^2^. The EQE spectra were measured through the Solar Cell Spectral Response Measurement System QE-R (Enlitech Co., Ltd.). TPC and TPV were obtained by the all-in-one characterization platform, Paios (Fluxim AG, Switzerland). In TPC testing, the light intensities were 10%, 17.8%, 31.6%, 56.2%, and 100% sunlight, respectively. The settling time was 100 µs, pulse length was 100 µs, and the follow-up time was 200 µs. In the TPV testing, the light intensities were 0.10%, 0.21%, 0.44%, 0.93%, 1.95%, 4.10%, 8.62%, 18.11%, 38.06%, and 80.0% sunlight, respectively. The settling time was 30 ms, pulse length was 5 ms, and the follow-up time was 30 µs. The conductivity of ETLs was estimated by measuring the current–voltage (*I*–*V*) curves of the devices with the structure of ITO/ETL/Ag using Ohm’s law (Fig. S1a). The pre-cleaned ITO substrates were treated with UV-ozone for 20 min. Then, the PDINN and D-PDINN were spin-coated on the ITO substrates. About 100 nm Ag was thermally evaporated under high vacuum. The *I*–*V* characteristics of these devices were measured in the dark. The electron and hole mobility was measured by the SCLC method, the electron-only devices were fabricated with the structure of ITO/ZnO/active layer/ETL/Ag and the hole-only devices were fabricated with the structure of ITO/PEDOT:PSS/active layer/MoO_3_/Ag. The mobility was calculated with the Mott–Gurney equation in the SCLC region:

$$J=\frac{9}{8}\varepsilon {\varepsilon }_{0}\mu \frac{{V}^{2}}{{L}^{3}}$$

where *J* is the current density, *ε*_0_ is the permittivity of free space, *ε*_r_ is the relative permittivity of the material,* L* is the thickness of the active layer, and *V* is the effective voltage. The *V*_TEF_ of ETL was also measured by SCLC method, using the device structure of Au/ETL/Au. Methanol solutions of PDINN or D-PDINN were spin-coated at a film thickness of ~ 5 nm on substrates with parallel Au electrodes (thickness = 100 nm) (Fig. S1b).

## Results and Discussion

### Preparation and Characterization of LiTFSI Doped PDINN

**Fig. 1:**
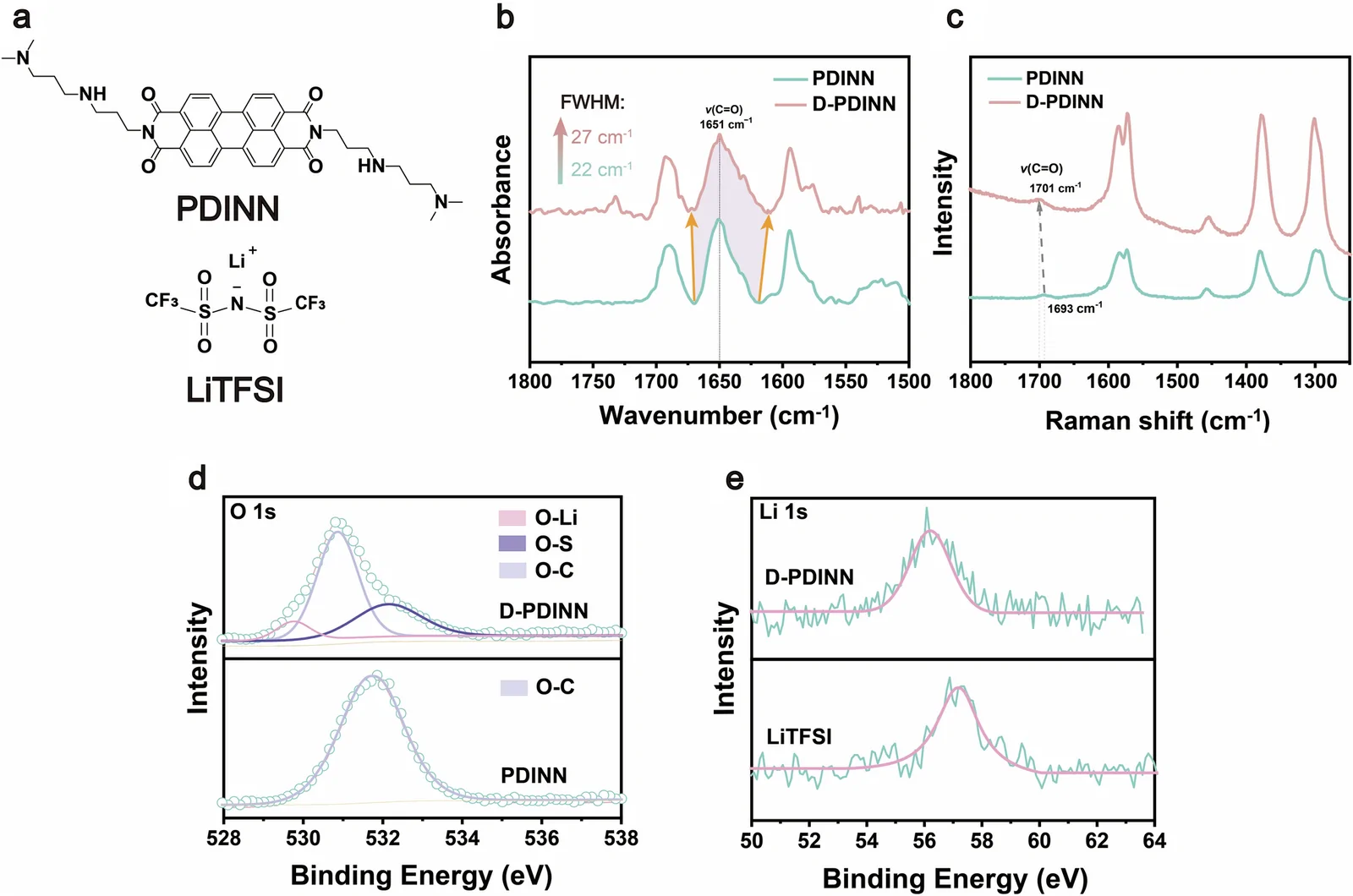
Interaction between LiTFSI and PDINN. **a** Molecular structures of PDINN and LiTFSI. **b** FT-IR spectra of PDINN and D-PDINN. **c** Raman spectra of PDINN and D-PDINN. **d** O 1*s* XPS spectra of PDINN and D-PDINN. **e** Li 1*s* XPS spectra of LiTFSI and D-PDINN

The molecular structures of PDINN [42] and LiTFSI are shown in Fig. 1a. The molecular geometries of PDINN, LiTFSI, and their adduct were investigated using density functional theory with ORCA 5.0.4 [43, 44] and visualized with VMD [45]. According to the electrostatic potential distributions of PDINN and LiTFSI (Fig. S2), the carbonyl oxygen in PDINN is negatively charged and the Li^+^ in LiTFSI is positively charged, allowing for a strong electrostatic attraction between them. Figure 1b shows the Fourier-transform infrared (FT-IR) spectra of PDINN and LiTFSI-doped PDINN (D-PDINN). The peak at 1651 cm^−1^ represents the stretching vibration of C=O bond in PDINN [42] which becomes boarder with the full width at half maximum (FWHM) increasing from 22 to 27 cm^−1^ after doping with LiTFSI, suggesting chemical environment around C=O has changed. Moreover, Raman spectra (Fig. 1c) show that the C=O peak blue-shifts from 1693 to 1701 cm^−1^ after the addition of LiTFSI, suggesting the electron density on C=O bond increases. X-ray photoelectron spectroscopy (XPS) was further employed to elucidate the interactions between PDINN and LiTFSI. The O 1*s* spectrum of PDINN shows a single peak at 531.7 eV (Fig. 1d), attributed to the binding energy of C–O binding [46]. As for D-PDINN, the O 1*s* peak can be decomposed into three peaks at 529.8, 530.9, and 532.1 eV, corresponding to Li–O, C–O, and S–O binding, respectively [47–49]. The binding energy shift of C–O peak indicates the chemical environment of carbonyl in PDINN changes after LiTFSI addition, suggesting carbonyl groups are the interaction sites in PDINN. The Li 1*s* XPS peak of LiTFSI locates at 57.2 eV (Fig. 1e), while that of D-PDINN shifts to 56.2 eV, implying Li^+^ participates in the interaction [46, 47]. Collectively, these results suggest that PDINN and LiTFSI interact through interaction between carbonyl and Li^+^.

**Fig. 2:**
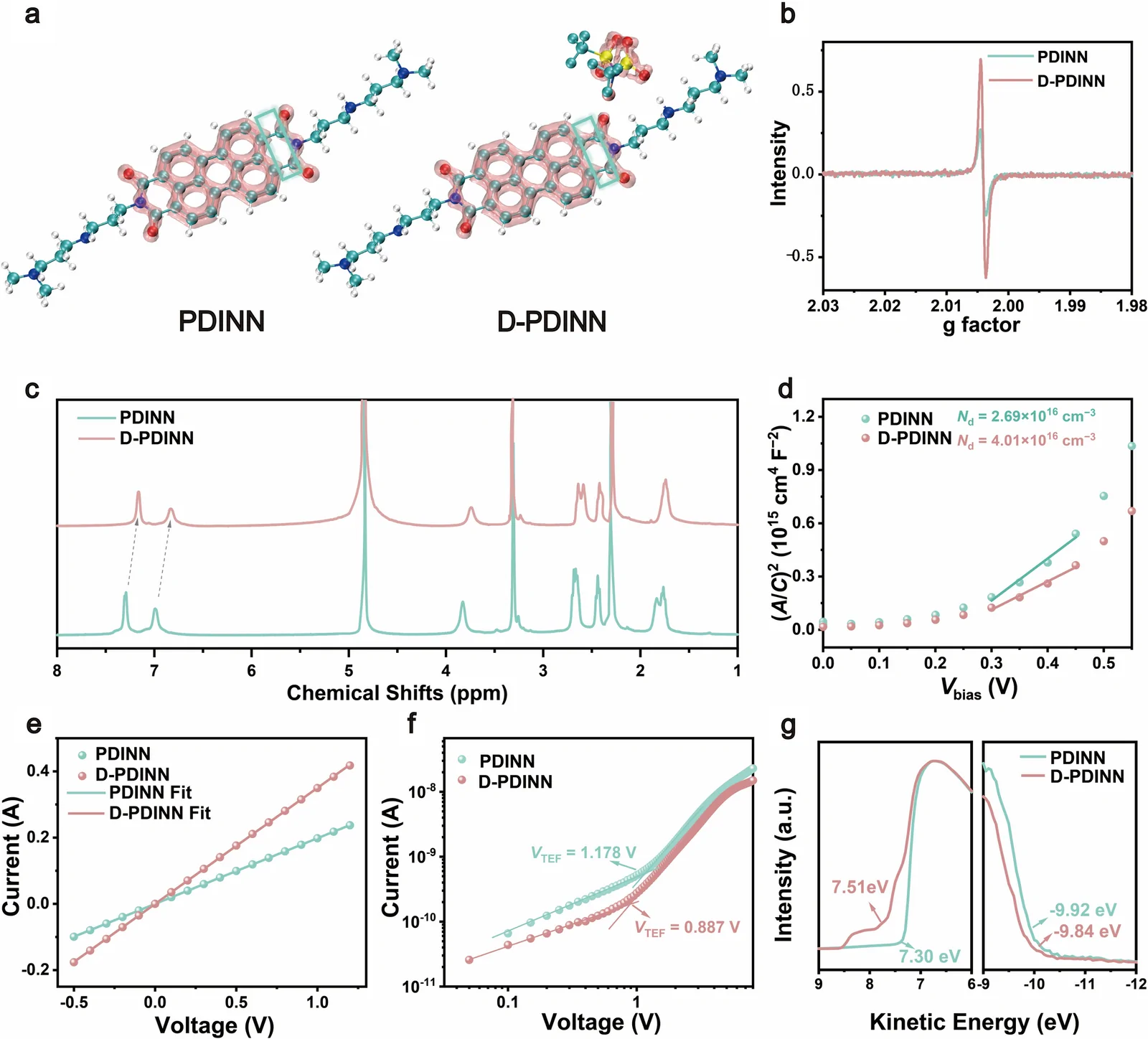
Intrinsic performance of PDINN and D-PDINN. **a** LOL-*π* isosurface of PDINN and D-PDINN with isovalue of 0.37. **b** Electron paramagnetic resonance (EPR) spectrum of PDINN and D-PDINN. **c** ^1^H HMR spectrum of PDINN and D-PDINN. **d** Mott–Schottky curves of ITO/PDINN or D-PDINN/Ag. **e** *I-V* curve for conductivity test. **f** SCLC curves of PDINN and D-PDINN with voltage increasing from 0 to 8 V. **g** UPS spectrum of Ag/PDINN and Ag/D-PDINN

To reveal the effects of LiTFSI doping, the *π*-electron delocalization of PDINN and D-PDINN was analyzed using localized orbital locator (LOL) with Multiwfn [50, 51]. At the same isovalue of 0.37, the LOL-*π* isosurface of PDINN is discontinued between the perylene and imide groups, while that of D-PDINN distributes across the perylene and an imide group, indicating the π electrons are more delocalized in D-PDINN (Fig. 2a). Electron paramagnetic resonance (EPR) spectroscopy is implemented to experimentally investigate the degree of electron delocalization. As shown in Fig. 2b, D-PDINN exhibits a stronger signal peak at the same position as PDINN with g factor of 2.004. Since the peak width is narrower than that of typical radicals, this peak is considered as a signal generated by the *π* electrons. The strengthened EPR signal in D-PDINN proves that LiTFSI induces more delocalized *π* electrons, which is beneficial to sizeable intermolecular *π*–*π* overlap and thereby enhanced the conductivity [52, 53]. Moreover, the *π*-delocalization is further investigated by ^1^H NMR (Fig. 2c). After the doping with LiTFSI, the peaks representing aromatic protons shift upfield: the peak at *δ* 7.29 ppm shifts to *δ* 7.17 ppm, and that at *δ* 6.99 ppm shifts to *δ* 6.83 ppm. The upfield shift indicates higher electron density around the protons, which suggests an increased electron delocalization in the aromatic system.

The doping density (*N*_d_) of PDINN and D-PDINN was measured with Schottky junction diodes and was calculated from the slope of the Mott–Schottky plot (Fig. 2d). The *N*_d_ of pure PDINN is 2.69 × 10^16^ cm^−3^, and after LiTFSI doping, D-PDINN shows an increased *N*_d_ of 4.01 × 10^16^ cm^−3^. For semiconductors, doping is one of the most effective strategies to increase charge carrier density and conductivity [53, 54]. *I–V* test was further conducted for ITO/PDINN or D-PDINN/Ag devices to determine the conductivity of the films (Fig. 2e). D-PDINN shows a higher conductivity of 1.71 × 10^−4^ S cm^−1^ than PDINN (9.67 × 10^−5^ S cm^−1^), confirming that the enhanced *π* delocalization, coupled with higher doping density, is beneficial to the improved conductivity.

The addition of LiTFSI to PDINN effectively reduces the trap density of the film. According to the space charge limited current (SCLC) test under dark conditions (Fig. 2f) and capacitance measurements at different frequencies (Fig. S3), the trap-filled-limited voltage (*V*_TFL_) and capacitance (*C*) of the D-PDINN are both lower than PDINN. According to the formula *N*_trap_ = 2*CV*_TFL_/*qdA* [55, 56] as the device area (*A*) and film thickness (*d*) remaining consistent between PDINN and D-PDINN, the lower *V*_TFL_ and *C* indicate that the trap density of the D-PDINN (7.26 × 10^16^ cm^−3^) is well suppressed compared to PDINN (1.29 × 10^17^ cm^−3^), which is beneficial to enhance charge extraction and suppress surface traps in STOPVs [57]. Additionally, atomic force microscopy was used to investigate the effects of LiTFSI on the morphology of PDINN (Fig. S4). The film surface becomes slightly smoother with root-mean-square roughness (*R*_*q*_) decreasing from 0.930 to 0.774 nm after the addition of LiTFSI, which is beneficial to reduce contact resistance and leakage current. And as shown in Fig. S5, the almost identical scanning electron microscopy of PDINN/Ag (12 nm) and D-PDINN/Ag (12 nm) implies the doping strategy will have no effect the AVTs of STOPVs.

The average lateral conductivity of PDINN/Ag (12 nm) and D-PDINN/Ag (12 nm) was (9.99 ± 0.30) × 10^4^ and (1.16 ± 0.03) × 10^5^ S cm^−1^ (Fig. S6), respectively, suggesting ETL doping can improve the lateral conductivity of electrode. The energy level adjustment ability after LiTFSI doping is measured by ultraviolet photoelectron spectroscopy (UPS) and cyclic voltammetry tests. As shown in Fig. 2g D-PDINN can effectively reduce the work function (*W*_F_) of the Ag/PDINN from 4.00 to 3.87 eV. The lower *W*_F_ implies a higher metal Fermi level, which can form better ohmic contact between active layer and electrode to accelerate collection efficiency. Moreover, D-PDINN processes lower LUMO (− 3.87 eV) and HOMO (− 5.84 eV) energy levels compared to PDINN (Fig. S7), which is beneficial to electron extraction and hole blocking.

### Photovoltaic Performance and Charge Carrier Dynamics

**Fig. 3:**
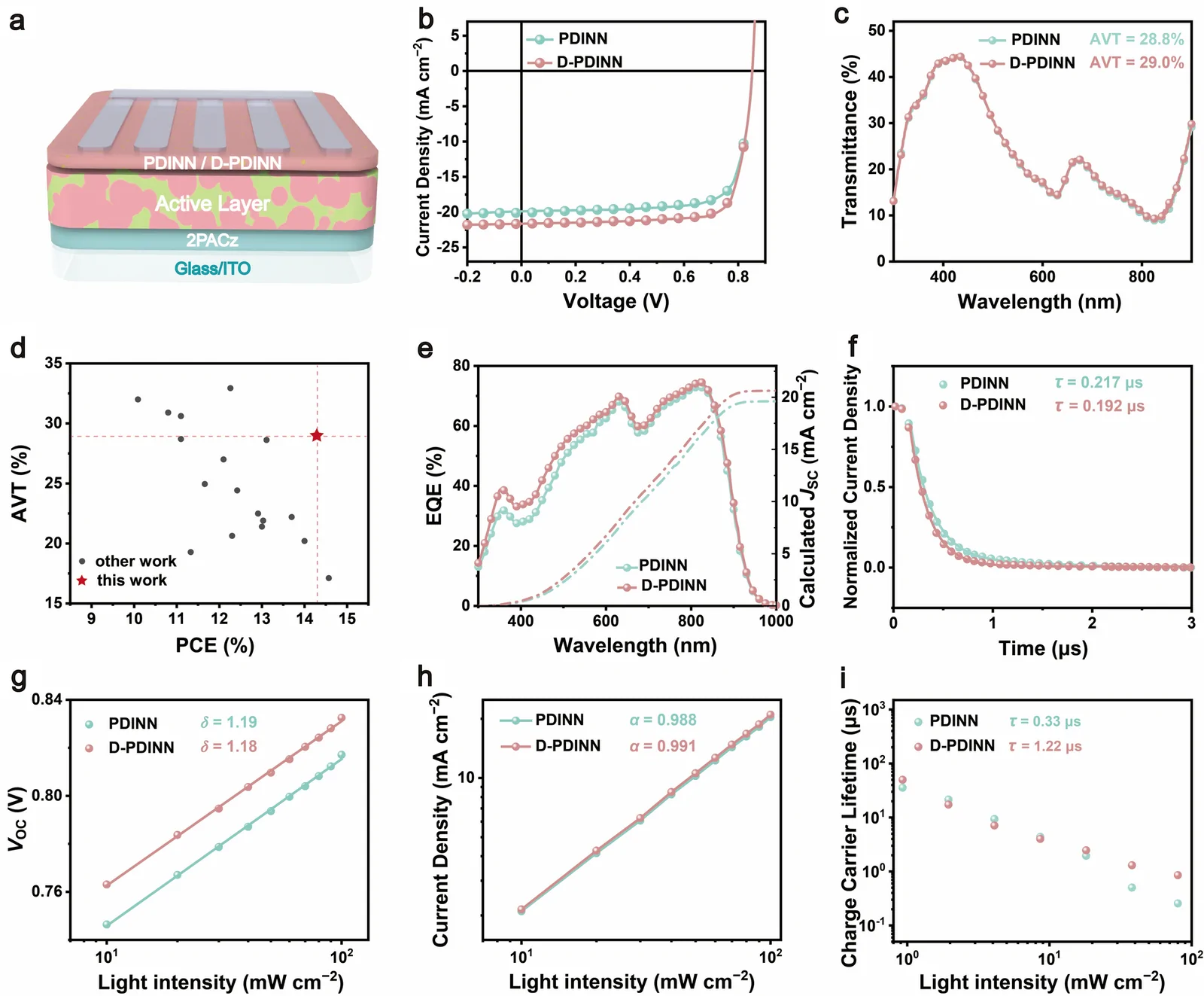
Photoelectrical performance of PDINN- and D-PDINN-based STOPVs. **a** Structure of STOPV device. **b** Transmittance spectra of the PDINN- and D-PDINN-based STOPVs. **c** *J–V* curves of the PDINN- and D-PDINN-based STOPVs. **d** Comparison of PCE and AVT value among modulation-free STOPVs. **e** EQE spectra of the PDINN- and D-PDINN-based STOPVs. **f** Normalized TPC curves of the PDINN- and D-PDINN-based STOPVs. **g** Charge carrier lifetimes under different Plight obtained from TPV experiment of the PDINN- and D-PDINN-based STOPVs. **h** Dependence of *V*_OC_ on* P*_light_ and **i** dependence of *J*_SC_ on *P*_light_ of the corresponding STOPVs

The structure of STOPV device is shown in Fig. 3a, and the molecular structures of used materials are shown in Fig. S8. The doping concentrations of LiTFSI in PDINN were first optimized. As the molar ratio of LiTFSI/PDINN changing from 0.01% to 100%, the AVTs of STOPVs do not differ much (Fig. S9a), while the PCEs are substantially influenced (Fig. S9b). At the optimal LiTFSI/PDINN molar ratio of 1%, the STOPVs exhibit a champion PCE of 14.3%, with *V*_OC_, *J*_SC,_ and FF of 0.846 V, 21.7 mA cm^−2^, and 78.3%, respectively, higher than the PDINN-based control STOPVs with a PCE of 13.0%, *V*_OC_ of 0.845 V, *J*_SC_ of 20.0 mA cm^−2^, and FF of 76.7% (Fig. 3b, Table 1). The AVT of PDINN- and D-PDINN-based devices is 28.8% and 29.0% (Fig. 3c). Therefore, the LUE, of D-PDINN-based STOPVs is enhanced to 4.15%, which is among the highest values of optical structure-free STOPVs (Fig. 3d, Table S1). The external quantum efficiency (EQE) spectra of the PDINN- and optimal D-PDINN-based STOPVs are shown in Fig. 3e. D-PDINN-based STOPVs exhibit a higher EQE response from 300 to 1000 nm, which results in a higher integrated *J*_SC_ of 20.6 mA cm^−2^ compared to PDINN-based devices (19.6 mA cm^−2^). The improvement of photovoltaic performance is also observed in opaque OPVs with device structure of ITO/2PACz/PM6:BTP-eC9/PDINN or D-PDINN/Ag (120 nm). The PDINN-based OPVs yield a PCE of 18.7% with *V*_OC_ of 0.844 V, *J*_SC_ of 28.7 mA cm⁻^2^ and FF of 77.0%. By doping PDINN with LiTFSI, a higher *J*_SC_ of 29.2 mA cm⁻^2^ is achieved, along with higher slightly higher *V*_OC_ of 0.846 V and FF of 78.3%, leading to improved PCE of 19.4% (Table S2, Fig. S10a). The EQE spectra confirm that D-PDINN leads to a greater photon response from 300 to 1000 nm than OPVs based on PDINN (Fig. S10b).

**Table 1:** Device data of STOPVs with PDINN or optimal D-PDINN as ETLs

ETL	*V*_OC_ (V)	*J*_SC_ (mA cm^−2^)	FF (%)	PCE (%)^*a*^	cal. *J*_SC_ (mA cm^−2^)^*b*^	AVT (%)^*c*^	LUE (%)
PDINN	0.845 (0.845 ± 0.001)	20.0 (20.3 ± 0.2)	76.7 (75.0 ± 0.9)	13.0 (12.9 ± 0.1)	19.6	28.8	3.74
D-PDINN	0.846 (0.846 ± 0.001)	21.7 (21.6 ± 0.2)	78.3 (78.1 ± 0.6)	14.3 (14.3 ± 0.1)	20.6	29.0	4.15

^*a*^Averaged values with standard deviation in parentheses were obtained from 5 devices

^*b*^*J*_SC_ calculated from the integration of EQE spectra with the AM 1.5G spectrum

^*c*^Arithmetic mean of transmittance in 400–700 nm

Besides, the LiTFSI doping is also effective to STOPVs based on other carbonyl-rich ETLs. Table S3 summarizes the key photovoltaic parameters of the STOPVs based on PDINO and PNDIT-F3N (Fig. S11). As for PDINO-based control STOPVs, the AVT is 27.8% and PCE is 13.1% with *J*_SC_ of 20.5 mA cm⁻^2^, *V*_OC_ of 0.833 V and FF of 76.6%. After doping PDINO with LiTFSI (D-PDINO), the AVT remains at 27.9%, while PCE improved to 13.7% with a higher *J*_SC_ of 21.3 mA cm⁻^2^, leading to a higher LUE of 3.96% (Fig. S12a, b). When doping PNDIT-F3N with LiTFSI (D-PNDIT-F3N), the STOPVs yield a higher PCE of 14.9% with higher *J*_SC_ of 23.0 mA cm⁻^2^ than PNDIT-F3N based control STOPVs with PCE of 13.7% and *J*_SC_ of 21.6 mA cm⁻^2^ (Fig. S13a). The AVTs of PNDIT-F3N and D-PNDIT-F3N-based STOPVs are 23.4% and 23.7% (Fig. S13b). As shown in Figs. S12c and S13c, D-PDINO and D-PDNIT-F3N lead to greater response of STOPVs in EQE spectra, which is consist with the improved *J*_SC_. The influence of doping strategy on PDINN- or D-PDINN-based STOPVs stability is investigated by storage under light (LED 100 mW cm^−2^), in the N_2_ atmosphere and ambient atmosphere (40% relative humidity), respectively. As shown in Fig. S14, the impact of LiTFSI doping strategy on the stability of STOPV devices is limited. 

The effects of LiTFSI doping on charge extraction were investigated by transient photocurrent (TPC) test (Fig. 3f). The charge extraction time of D-PDINN-based STOPVs is 0.192 μs, shorter that of PDINN-based STOPVs (0.217 μs). The faster charge extraction in D-PDINN-based STOPVs benefits higher *J*_SC_ and FF, which can be attributed to the improved conductivity relative to pristine PDINN. Light intensity (*P*_light_) dependence of *V*_OC_ and *J*_SC_ was used to characterize the charge recombination in the devices. The equation of *V*_OC_ *∝* (*δkT*/*q*) ln (*P*_light_) describes the relationship between *V*_OC_ and *P*_light_, where *k*, *T*, and *q* are the Boltzmann constant, temperature, and elementary charge, respectively. The *δ* represents whether the process is dominated by bimolecular recombination (*δ* → 1) or trap-assisted recombination (*δ* → 2). The *δ* values are calculated to be 1.19 for the PDINN-based STOPVs and 1.18 for the D-PDINN-based STOPVs (Fig. 3g), indicating that the trap-assisted recombination is slightly suppressed in D-PDINN-based devices. The* J*_SC_–*P*_light_ dependence follows the relationship of *J*_SC_ *∝* *P*_light_^*α*^, where *α* represents the degree of bimolecular recombination and *α* closer to 1 means less bimolecular recombination. The D-PDINN-based STOPVs exhibit a higher *α* of 0.991 than PDINN-based counterparts (0.988) (Fig. 3h), indicating that bimolecular recombination is suppressed in the D-PDINN-based STOPVs. The weaker bimolecular recombination could be originated from the less charge carrier accumulation at the interface between active layer and ETL due to the enhanced conductivity of D-PDINN. The charge carrier lifetime (*τ*) in devices was extracted from transient photovoltage (TPV) experiments (Fig. 3i). The *τ* of D-PDINN-based devices is 1.22 μs, longer than that of 0.33 μs in PDINN-based ones, suggesting less charge recombination in D-PDINN-based STOPVs, consistent with the results from *V*_OC_–*P*_light_ and* J*_SC_–*P*_light_ analysis.

The light-to-electricity conversion processes in devices were evaluated by measuring the photocurrent density (*J*_ph_) under different effective voltage (*V*_eff_) (Fig. S15a). At high *V*_eff_ (> 2.90 V), all generated charges are swept out, and the *J*_ph_ becomes saturated photocurrent density (*J*_sat_). The *J*_sat_ of PDINN- and D-PDINN-based devices are 20.2 and 21.89 mA cm^−2^, respectively. The ratio of *J*_ph_/*J*_sat_ represents the overall efficiency of the light-to-electricity conversion at different *V*_eff_ (Fig. S15b). D-PDINN-based devices show higher *J*_ph_/*J*_sat_ than PDINN-based devices, especially at low *V*_eff_ region. Since the active layers are the same, the exciton generation, charge separation, and charge transport can be assumed identical in these devices at a fixed *V*_eff_. Therefore, the higher *J*_sat_ indicates more charges can be output due to the suppressed charge accumulation at the active layer/ETL interface and thereby less charge recombination during charge extraction process; and the higher *J*_ph_/*J*_sat_ suggests the charge extraction in D-PDINN-based devices is more efficient, which is beneficial to higher *V*_OC_, *J*_SC_, and FF.

### Quantitative Analysis for Recombination Current

**Fig. 4:**
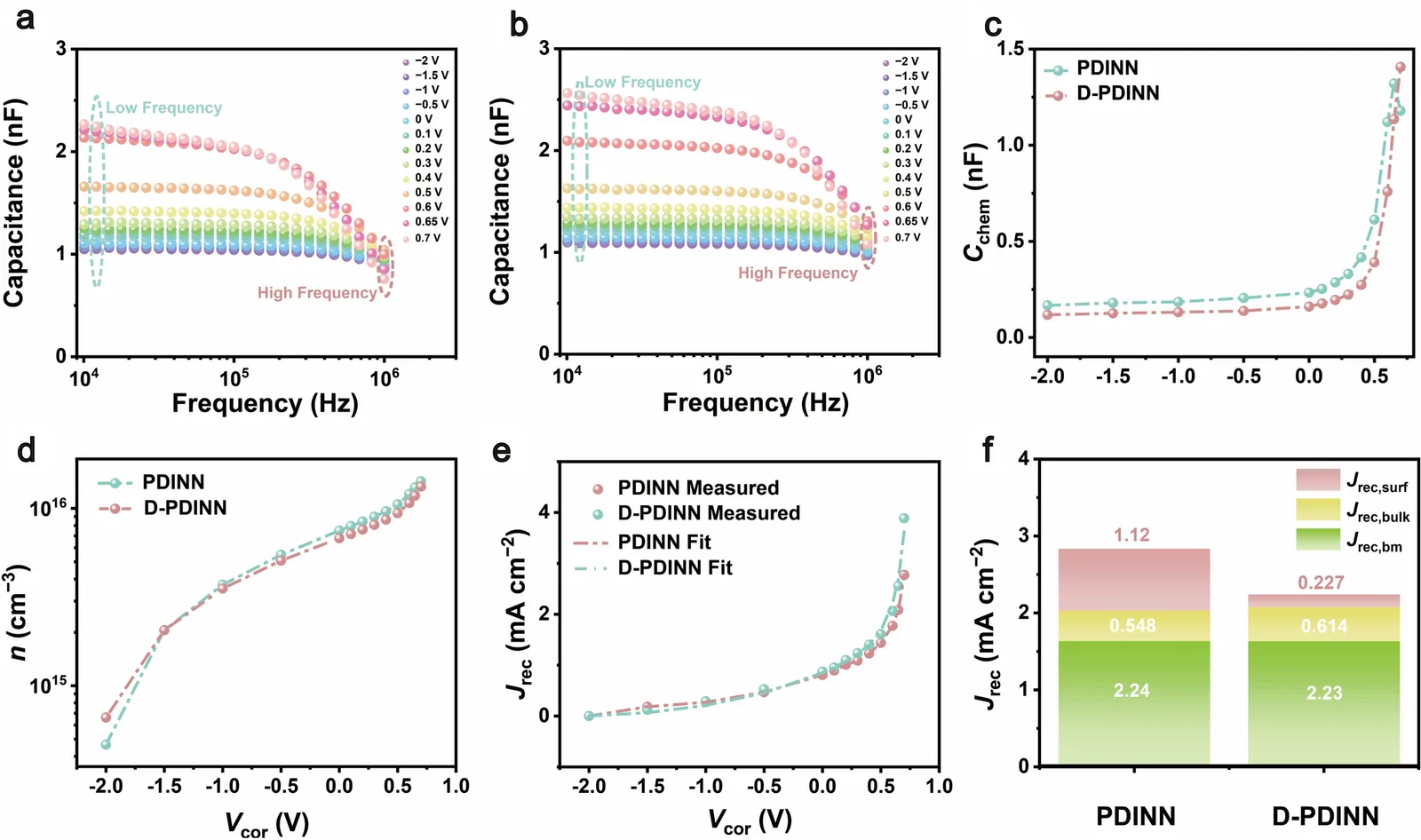
Capacitance of **a** PDINN- and **b** D-PDINN-based STOPVs at different biases under the AM 1.5G illumination (100 mW cm^−2^). **c** Chemical capacitance of the STOPVs based on PDINN and D-PDINN. **d** Charge carrier density of PDINN- and D-PDINN-based STOPVs. **e** Measured total recombination current densities and their fits for STOPVs based on PDINN and D-PDINN. **f** Histograms of recombination current densities for PDINN- and D-PDINN-based STOPVs

The surface trap-assisted recombination is a major cause of the electrical loss in STOPVs [34], and the effects of LiTFSI doping on the traps and recombination current were investigated using capacitance spectroscopy [34, 58, 59]. To begin with, the capacitance of PDINN (Fig. 4a) and D-PDINN-based devices (Fig. 4b) was measured under the AM 1.5G irradiation at different biases and in the dark (Fig. S16a), from which the chemical capacitance (*C*_chem_) (Fig. 4c) can be obtained to calculate the density of free charge carriers (*n*) (Fig. 4d) with *V*_bi_ from Mott–Schottky curves (Fig. S16b) and electron and hole mobility from SCLC tests (Fig. S17). The recombination current density (*J*_rec_) is determined from *J*-*V* curves and can be fitted as the sum of bimolecular recombination current density (*J*_rec,bm_), bulk trap-assisted recombination current density (*J*_rec,buk_), and surface trap-assisted recombination current density (*J*_rec,surf_), using bimolecular recombination coefficient (*ξ*), bulk trap density (*N*_t,bulk_), and surface trap density (*N*_t,surf_) as fitting parameters (Fig. 4e). The fitting details are presented in Supporting Information. PDINN-based devices show a *ξ* of 0.0127, *N*_t, bulk_ of 2.22 × 10^14^ cm^−3^, and *N*_t, surf_ of 5.01 × 10^10^ cm^−2^, while D-PDINN-based devices show a lower *ξ* of 0.0120, *N*_t, bulk_ of 1.34 × 10^14^ cm^−3^, and *N*_t, surf_ of 8.66 × 10^9^ cm^−2^ (Table 2). The lower *ξ* suggests weaker bimolecular recombination, and lower *N*_t, bulk_ and *N*_t, surf_ correspond to suppressed trap-assisted recombination. Notably, D-PDINN-based STOPVs exhibit a 82.7% reduction in *N*_t, surf_, which indicates the surface traps are effectively suppressed, contributing to lower photocurrent losses in STOPVs. Figure 4f shows the *J*_rec, bm_, *J*_rec, buk_, and *J*_rec, surf_ in the STOPVs. In comparison with PDINN-based STOPVs, D-PDINN-based STOPVs showed slight suppression in *J*_rec, bm_ and* J*_rec, bulk_. In contrast, the reduction in *J*_rec, surf_ is more pronounced, reducing from 1.12 mA cm^−2^ in PDINN-based STOPVs to 0.227 mA cm^−2^ in D-PDINN-based ones. These results support the effectiveness of the LiTFSI doping strategy in inhibiting surface trap-assisted recombination, which is a primary cause of photocurrent loss in STOPVs, ultimately promoting the performance of STOPVs.

**Table 2:** Fitting parameters of the recombination current densities of PDINN- and D-PDINN-based STOPVs

ETL	*ξ*	*N*_t, bulk_ (cm^−3^)	*N*_t, surf_ (cm^−2^)
PDINN	0.0127	2.22 × 10^14^	5.01 × 10^10^
D-PDINN	0.0120	1.34 × 10^14^	8.66 × 10^9^

## Conclusions

In summary, we present an effective strategy to enhance the charge collection ability and conductivity of the ultrathin Ag electrode in STOPVs via ETL doping. XPS and FT-IR results reveal that LiTFSI interacts with PDINN through the electrostatic attraction between Li^+^ and carbonyl. Owing to this interaction, the π electrons in PDINN become more delocalized, as evidenced by the stronger EPR signal. As a result, relative to PDINN, D-PDINN shows increased conductivity from 9.67 × 10^−5^ to 1.71 × 10^−4^ S cm^−1^, increased doping density from 2.69 × 10^16^ to 4.01 × 10^16^ cm^−3^, and decreased trap density from 1.29 × 10^17^ to 7.26 × 10^16^ cm^−3^. The *W*_F_ of D-PDINN decreases to 3.87 eV compared to 4.00 eV for PDINN/Ag, contributing to better ohmic contact between active layer and electrode. The optimized D-PDINN also provides lateral conductive channel for discontinuous ultrathin Ag electrode, improving its conductivity and charge collection ability. Consequently, D-PDINN-based STOPVs exhibit enhanced performance compared to PDINN-based counterparts, with PCE increased from 13.0% to 14.3%, *J*_SC_ from 20.0 to 21.7 mA cm^−2^, FF from 76.9% to 78.3%, and LUE from 3.74% to 4.15%. Charge dynamics in the devices were investigated by TPC, TPV, SCLC, light dependent test of *J*_SC_ and *V*_OC_ and capacitance spectroscopy, which demonstrates that D-PDINN-based STOPVs exhibit enhanced electron extraction, suppressed surface trap density and lower surface trap-assisted recombination current. This work provides a new mechanism of manipulating π-delocalization of ETL to optimize the electrical properties of ultrathin metal electrode and highlights the potential of interfacial engineering for efficient STOPVs. Based on this discovery, the future investigation can focus on exploring diverse cations with different valencies or ionic radii on and their influence on the properties of organic semiconductors.

## Supplementary Information

Below is the link to the electronic supplementary material.

Supplementary file 1 (DOCX 2133 KB)
