# Supplementary material for: Enhancing π-Delocalization and Suppressing Traps via Doping in Electron Transport Materials for Efficient Semitransparent Organic Photovoltaics
Source: Nanomicro Lett. 2026 Feb 9;18:247. doi: 10.1007/s40820-026-02083-1 (PMC12886666; doi:10.1007/s40820-026-02083-1)
Supplement: Supplementary file 1 — (DOCX 2133 KB) [file 40820_2026_2083_MOESM1_ESM.docx]

Supporting Information for

**Enhancing π-Delocalization and Suppressing Traps via Doping in Electron Transport Materials for Efficient Semitransparent Organic Photovoltaics**

Yating Mo^1^, Jiayu Wang^1,^*, Hanjiao Chen^2^, Yufei Gong^3^, Jianglong Zhou^4^, Junhao Lu^1^, Cenqi Yan^1^, Lei Meng^3^, Liang-Wen Feng^4^, Yongfang Li^3^ and Pei Cheng^1,^*

^1^ College of Polymer Science and Engineering, State Key Laboratory of Advanced Polymer Materials, Sichuan University, Chengdu, 610065, People’s Republic of China

^2^ Analytic & Testing Center, Sichuan University, Chengdu, 610065, People’s Republic of China

^3^ Beijing National Laboratory for Molecular Sciences, CAS Key Laboratory of Organic Solids, Institute of Chemistry, Chinese Academy of Sciences, Beijing 100190, People’s Republic of China

^4^ Key Laboratory of Green Chemistry & Technology, Ministry of Education, College of Chemistry, Sichuan University, Chengdu 610065, People’s Republic of China

*Corresponding authors. E-mail: wangjiayu@scu.edu.cn (Jiayu Wang); chengpei@scu.edu.cn (Pei Cheng)

**S1 Experimental Section**

**S1.1 Impedance measurement**

The tests were conducted on Gamry reference 620 Potentiostat. The dielectric constant was evaluated from the geometric capacitance (*C*_g_), which represents the capacitance derives from only the material itself. The frequency-dependent capacitance (*f*-*C*) spectrum of the parallel plate capacitor is measured at a reverse bias of −2 V in the dark, and the *C*_g_ is extracted at a frequency of 10^6^ Hz. The dielectric constant can be calculated according to the following equation:

$$\varepsilon_{r}=\frac{C_{g}L}{\varepsilon_{0}A}$$

where *L* is the thickness of the film, *ε_0_* is the permittivity of the vacuum and *A* is the device area. The doping density (*N*_d_) is calculated according to the Mott-Schottky analysis. The device structure was ITO/ETL/Ca (10 nm)/Ag (100 nm). The capacitance-voltage (*C*-*V*) characteristics were measured at the range of −2 V ~ 0.9 V under an AC signal (10^5^ Hz). The *N*_d_ is calculated according to the following equation:

$$N_{d}=(\frac{2}{q\varepsilon_{0}\varepsilon_{r}}){(\frac{d(\frac{A^{2}}{C^{2}})}{dV})}^{-1}$$

where *e* is elementary charge, *ε*_0_ is the permittivity of vacuum and *ε*_r_ is dielectric constant. The impedances of the devices were measured under AM 1.5G 100 mW cm^−2^ illumination, and different biases and an AC signal with an amplitude of 50 mV were applied to the devices during the measurement. The capacitance (*C*_b_) is calculated from impedance measurements using the following equation:

$$C_{b}=-\frac{1}{\omega}\left[ \frac{Z^{''}-\omega L}{\left( Z^{'}-R_{s} \right)^{2}+\left( Z^{''}-\omega L \right)} \right]$$

where is *ω* the angular frequency, $Z^{'}$ and $Z^{''}$ are the real and imaginary components of the measured impedance, *L* = 5 × 10^−6^ H is the parasitic inductance of the connected wires and *R*_S_ is the series resistance of the solar cell.

The chemical capacitance *C*_chem_ can be determined as the difference between *C*_b_ at low (LF) and high (HF) frequency:

$$\text{C}_{\text{chem}}=\text{C}_{\text{LF}}-\text{C}_{\text{HF}}$$

The density of charge carriers (*n*) induced by light can be determined by the following equation:

$$n\left( V_{cor} \right)=\frac{1}{qAL}C_{sat}(V_{0}-V_{sat})+\frac{1}{qAL}\int_{V_{sat}}^{V_{cor}} C_{chem}dV_{cor}$$

where *V*_sat_ is the reverse bias voltage at saturated photocurrent (−2V), *n*_sat_ is the charge carrier density at *V*_sat_, *A* is the area of the solar cell, *C*_sat_ is the difference in capacitance of the STOPVs under illumination and dark conditions at *V*_sat_, *V*_0_ is the forward bias when photocurrent equals to zero.

The recombination current density *J*_rec_ can be determined as follows:

$$J_{rec}=J_{sat}-J_{ph}=J_{ph,sat}-(J_{light}-J_{dark})$$

where *J*_ph,sat_ is the saturated photocurrent density, usually extracted at a reverse bias (−2V), and *J*_ph_ is the photocurrent density, defined as the difference between the current density under illumination (*J*_light_) and in the dark (*J*_dark_).

The *J*_rec_ stems from bimolecular (*J*_rec,bm_) and trap-assisted recombination, and the trap states distribute in bulk (*J*_rec,buk_) and surface (*J*_rec,surf_), which were quantitatively described as follows:

$$J_{rec,bm}=qLk_{bm}n^{2}=\frac{q^{2}L}{\varepsilon_{0}\varepsilon_{r}}\xi(\mu_{e}+\mu_{h})n^{2}$$

$$J_{rec,bulk}=qLk_{bulk}n=\frac{q^{2}L}{\varepsilon_{0}\varepsilon_{r}}\mu_{e}N_{t,bulk}n$$

$$J_{rec,surf}=qLk_{surf}n=\frac{q^{2}}{\varepsilon_{0}\varepsilon_{r}}\frac{\mu_{h}N_{t,surf}n}{exp\left\{ \frac{q(V_{bi}-V_{cor})}{kT} \right\}}$$

*q* is the elementary charge; L is the thickness of the active layer; *k*_bm_, *k*_bulk_, and *k*_surf_ are the bimolecular, bulk-trap assisted, and surface-trap-assisted recombination coefficients, respectively; *n* is the density of free charge carriers; *ε*_0_ is the vacuum dielectric constant; *ε*_r_ is the dielectric constant of the active layer; *V*_bi_ is the built in voltage of device, which is calculated using the Mott-Schottky model by plotting *A*^2^/*C*^2^ with *V*_cor_ (Fig. S9b); $\mu_{e}$and$\mu_{h}$ are electron and hole mobilities (Fig. S10); *V*_cor_ is the corrected voltage (*V*_cor_ = *V*_applied_ $-$*J×R*_series_); *k* is the Boltzmann constant; *T* is the temperature; $\xi$ is the Langevinpre factor; *N*_t,bulk_ is the density of traps in the bulk; and *N*_t,surf_ is the density of surface traps, which are important parameters to quantify the bimolecular, bulk-trap-assisted, and surface-trap-assisted recombination, respectively

**S1.2 Molecular simulation**

Density functional theory (DFT) calculations were performed with the ORCA 5.0.4^[S1, S2]^ program using the B3LYP functional^[S3, S4]^ and visualized by VMD.^[S5]^ All-electron split valence basis set with polarization functions def2-SVP was used for all atoms.^[S6]^ Geometry optimizations were performed with full relaxation of all atoms in gas phase without solvent effects. The vibrational frequency calculations were performed to check that the stable structures had no imaginary frequency. The delocalization of π electrons was analyzed using Multiwfn.^[S7]^

**S1.3 ETL characterization and analysis**

FT-IR spectra were recorded using a Nicolet 6700 spectrometer covering the IR frequency range from 4000 cm^−1^ to 400 cm^−1^. Raman spectroscopy was conducted for Cu/PDINN and Cu/D-PDINN on a Renishaw inVia Reflex Raman spectrometer. The PDINN and D-PDINN films were spined by methanol solution (10mg mL^−1^) at 800rpm. XPS were conducted on AXIS Ultra DLD photoelectron spectrometer. The UV-vis transmission spectra were conducted on a Shimadzu UV-2600i spectrophotometer. Cyclic voltammetry was measured in anhydrous acetonitrile solution with 0.1 mol L^−1^ Bu_4_NPF_6_ using electrochemical workstation (CHI660) with a three-electrode cell at a scan rate of 50 mV s^−1^. A Pt wire, a glassy carbon electrode and Ag/AgCl electrode were used as the counter electrode, the working electrode and the reference electrode. Potentials were referenced to ferrocene/ferrocenium redox couple (Fc/Fc+, −4.8 eV) as external standard. EPR was conducted on a Bruker EMXplus X-band EPR spectrometer at room temperature. PDINN or D-PDINN was dissolved in methanol (10mg mL^−1^), and the frequency and the power of microwave were 9.86 GHz and 2.00 mW, respectively. ^1^H HMR measurements were conducted on a Bruker AV III HD 400 MHz NMR spectrometer for PDINN and D-PDINN dissolved in CD₃OD (5mg mL^−1^). UPS measurements were conducted on Kratos Axis Ultra DLD photoelectron spectrometer with He I source (21.22 eV) under an applied negative bias of 10.0 V. based on Ag/PDINN or Ag/D-PDINN. The conductivity of Ag (12 nm)/PDINN or Ag (12 nm)/D-PDINN was measured by four-terminal sensing on FT-371A.

**S2 Supplementary Figures**


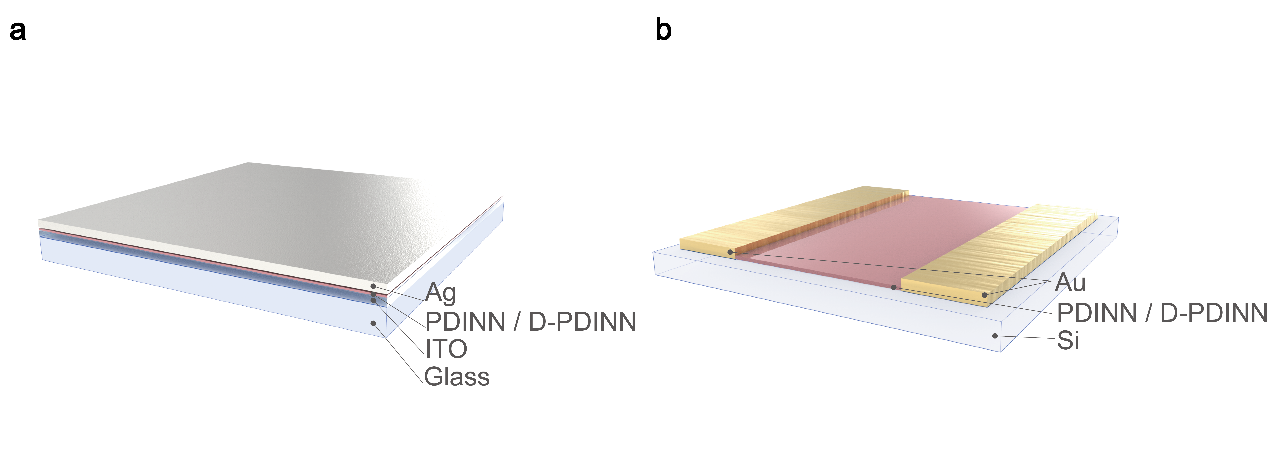


**Fig. S1** (**a**) Vertical device structure of ITO/PDINN or D-PDINN/Ag for *I*–*V* tests. (**b**) Lateral device structure of Au/PDINN or D-PDINN/Au for SCLC tests


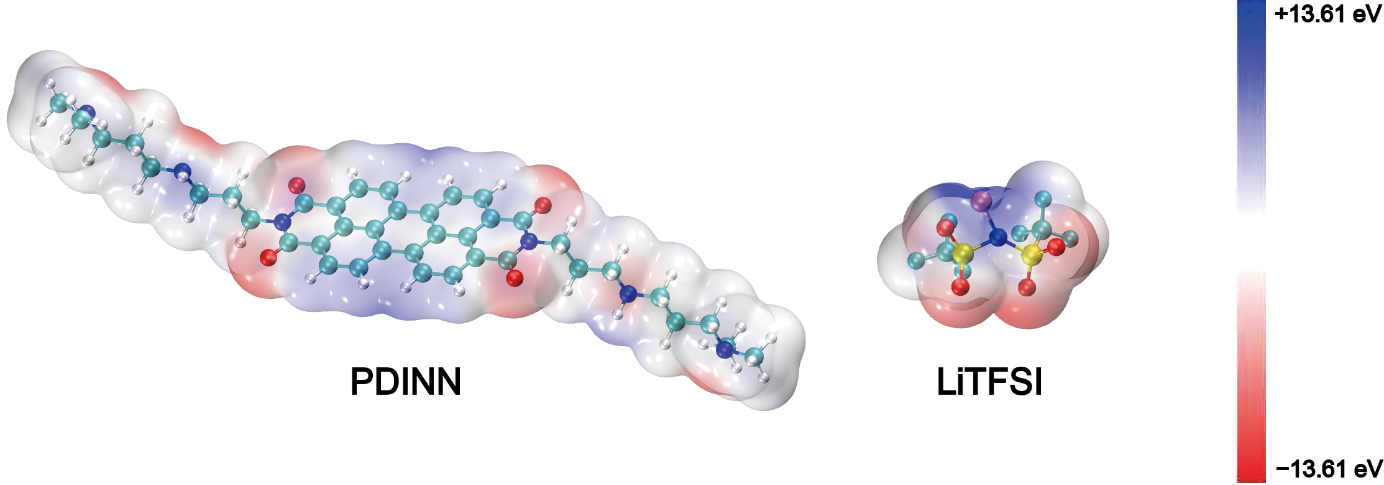


**Fig. S2** Electrostatic potential distributions of PDINN and LiTFSI


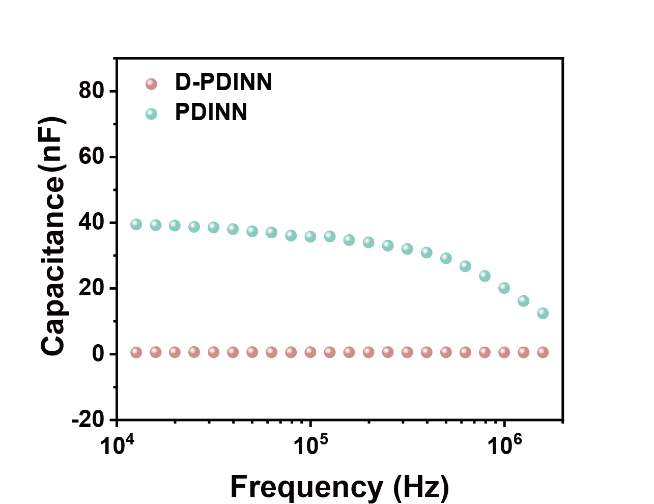


**Fig. S3** The frequency dependent capacitance of ITO/PDINN/Ca/Ag and ITO/D-PDINN/Ca/Ag


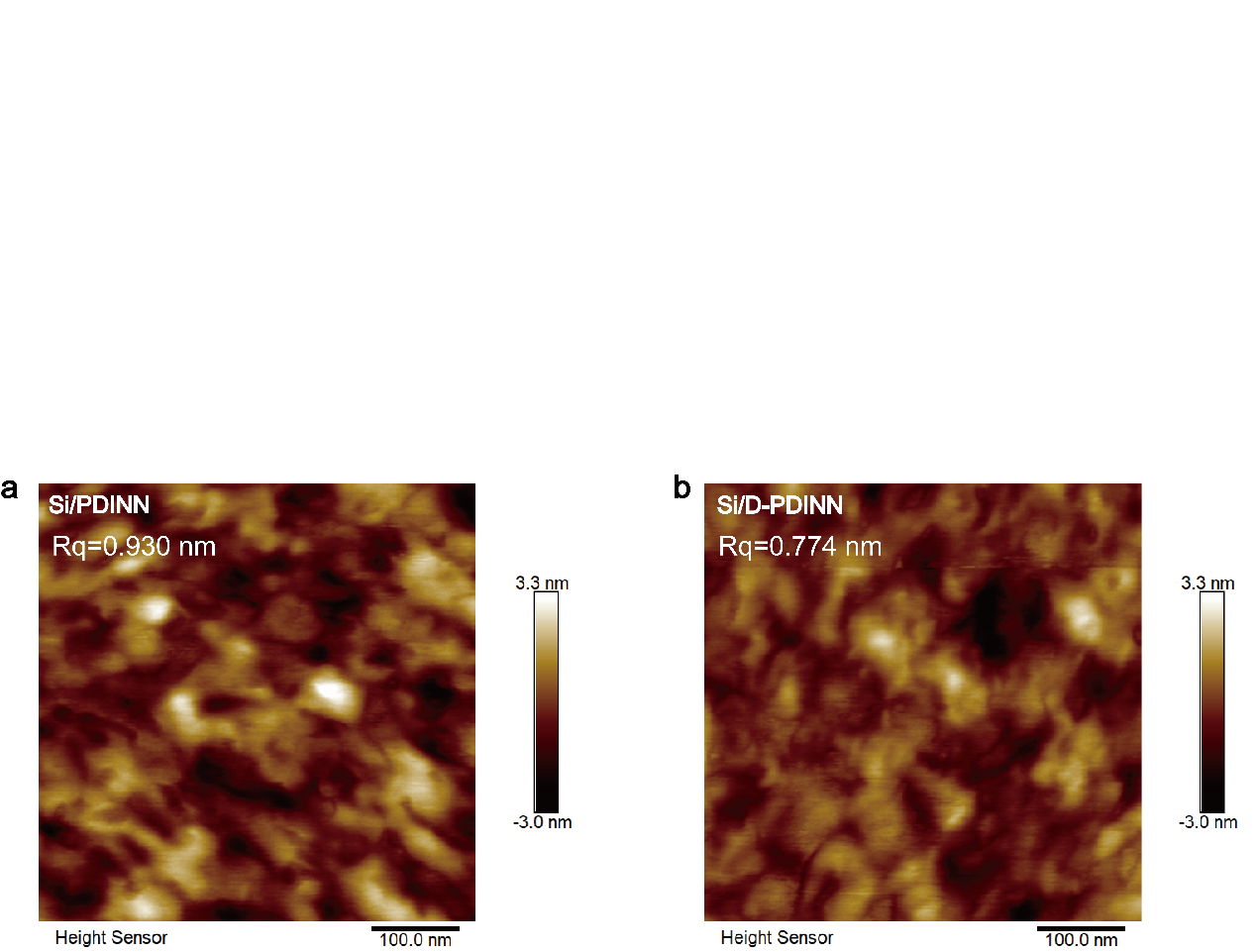


**Fig. S4** The atomic force microscopy images of (**a**) PDINN and (**b**) D-PDINN


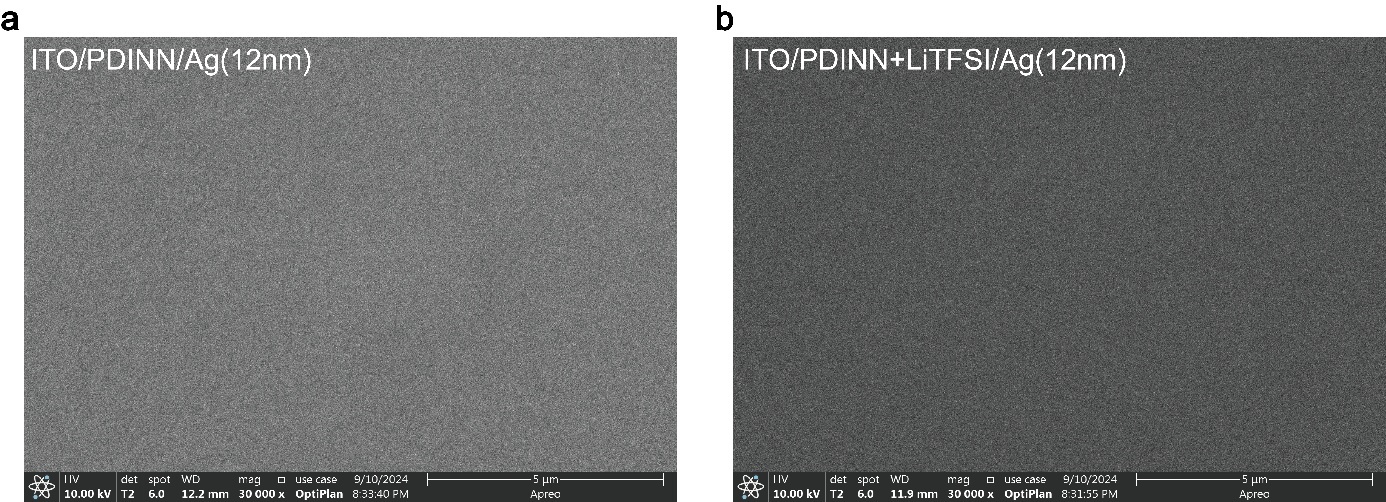


**Fig. S5** The scanning electron microscopy images of (**a**) PDINN/Ag and (**b**) D-PDINN/Ag


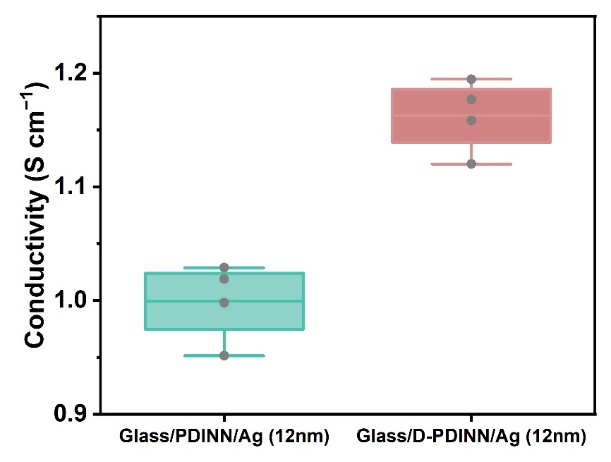


**Fig. S6** The statistical analysis plot of lateral conductivity of glass/PDINN or D-PDINN/Ag (12 nm) from 4 independent measurements


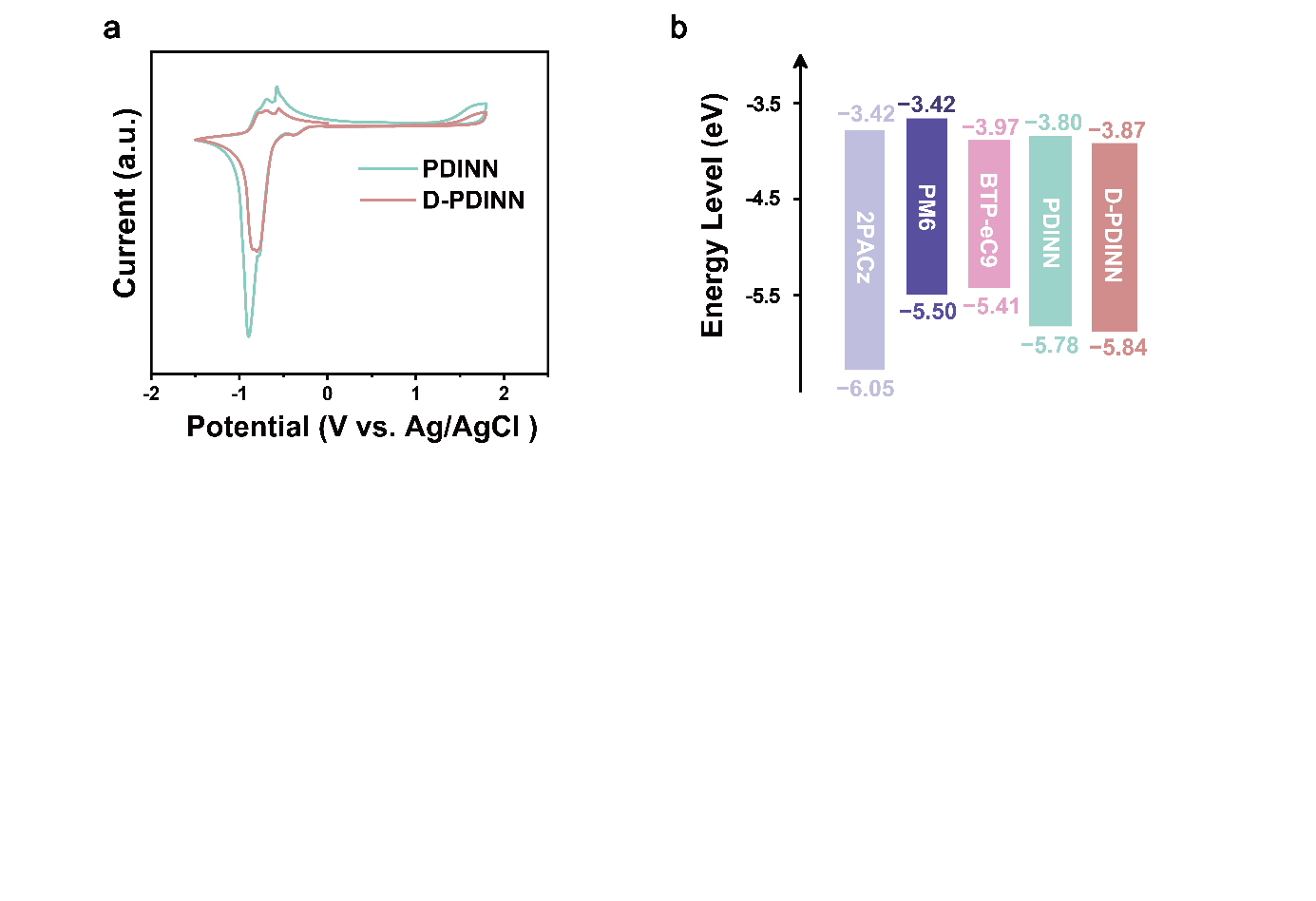


**Fig. S7** (**a**) Cyclic voltammetry curves of PDINN and D-PDINN. (**b**) Energy level diagram of PDINN, D-PDINN, 2PACz, PM6 and BTP-eC9


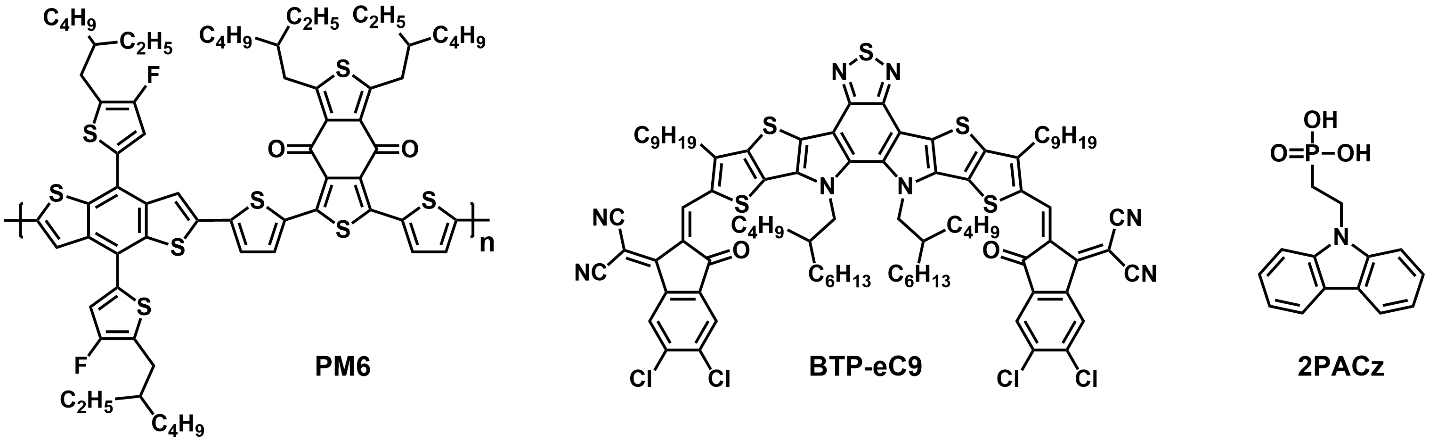


**Fig. S8** Molecular structures of PM6, BTP-eC9 and 2PACz


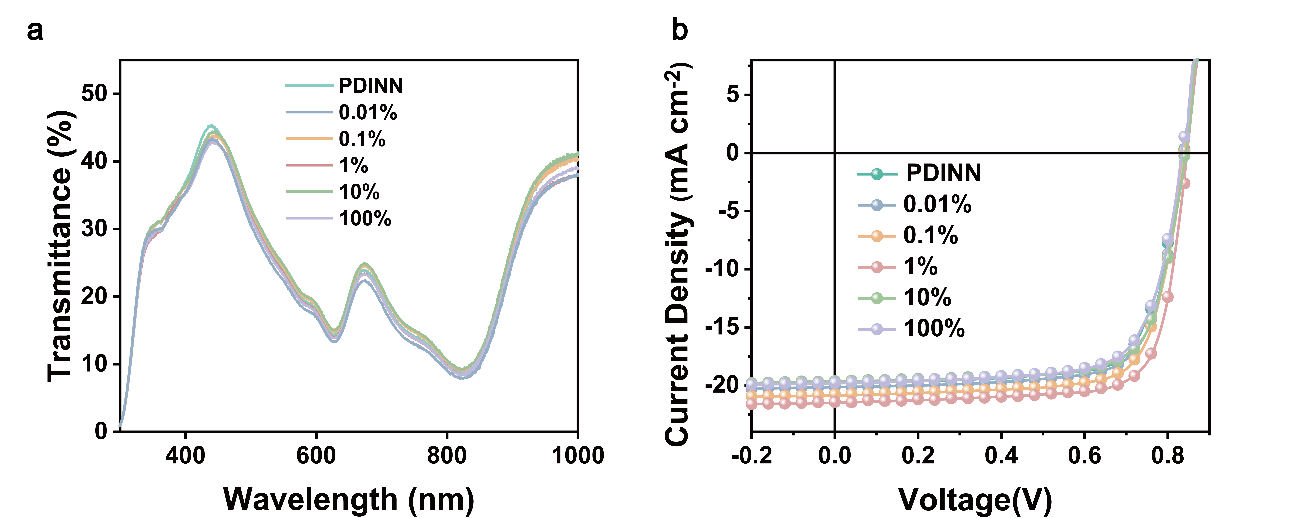


**Fig. S9** (**a**) The transmittance spectra. (**b**) The *J*–*V* curves of STOPVs based on PDINN and LiTFSI:PDINN with different molar ratio


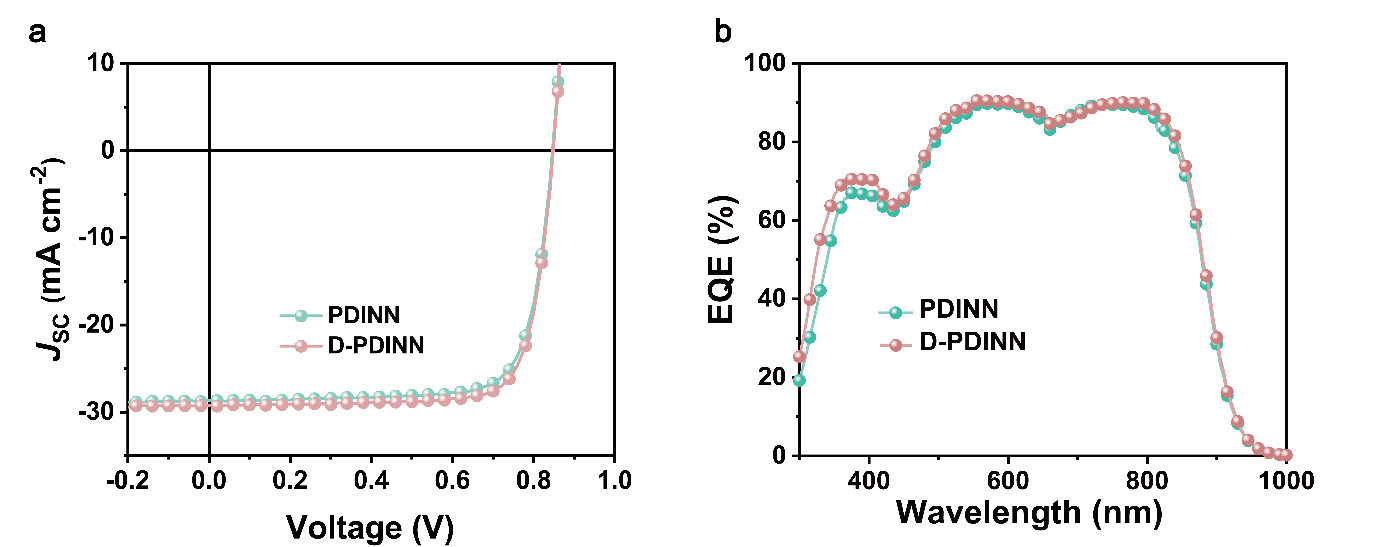


**Fig. S10** (**a**)The *J*–*V* curves and (**b**) EQE spectra of opaque OPVs based on PDINN and D-PDINN


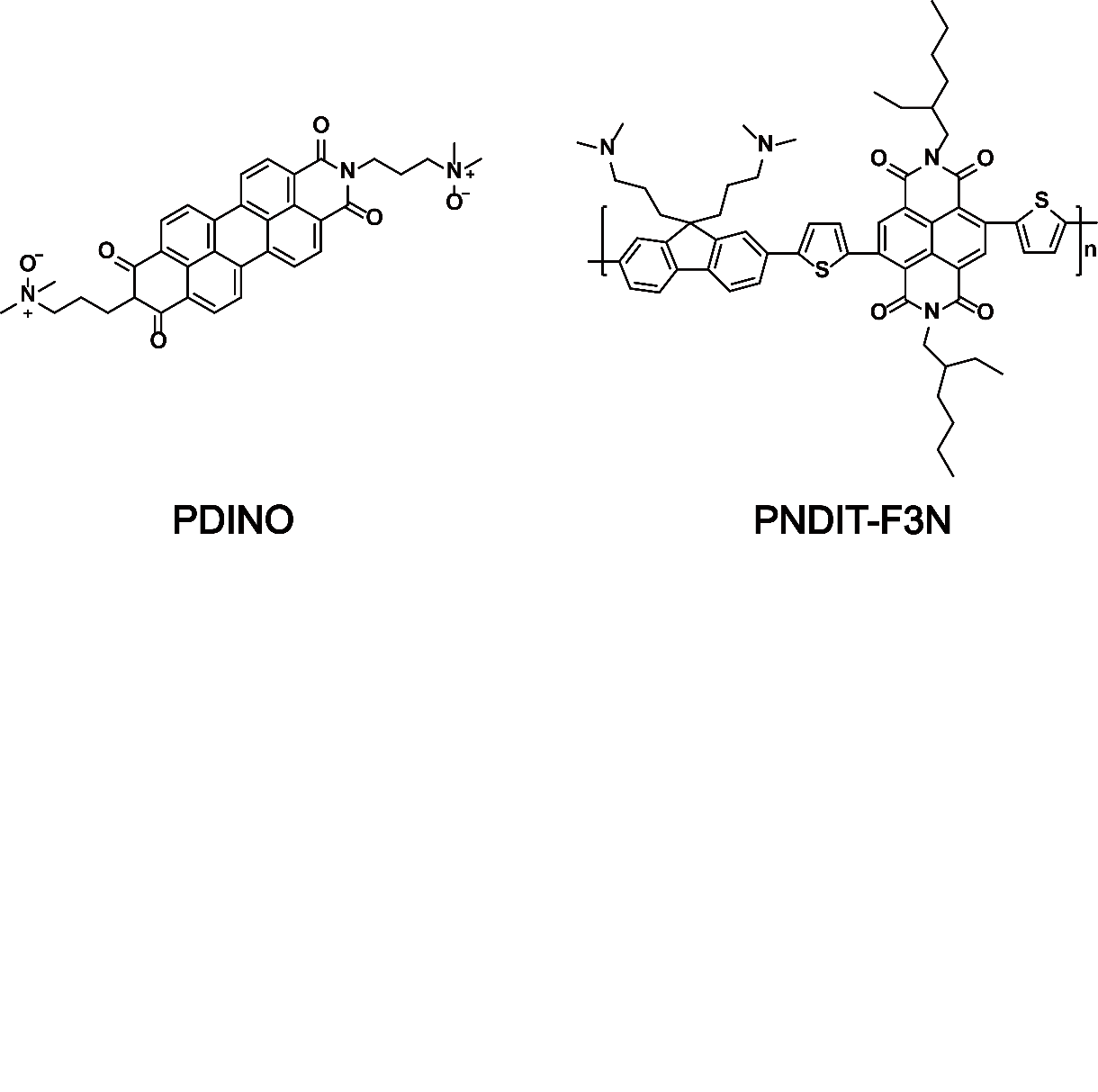


**Fig. S11** Molecular structures of PDINO and PNDIT-F3N


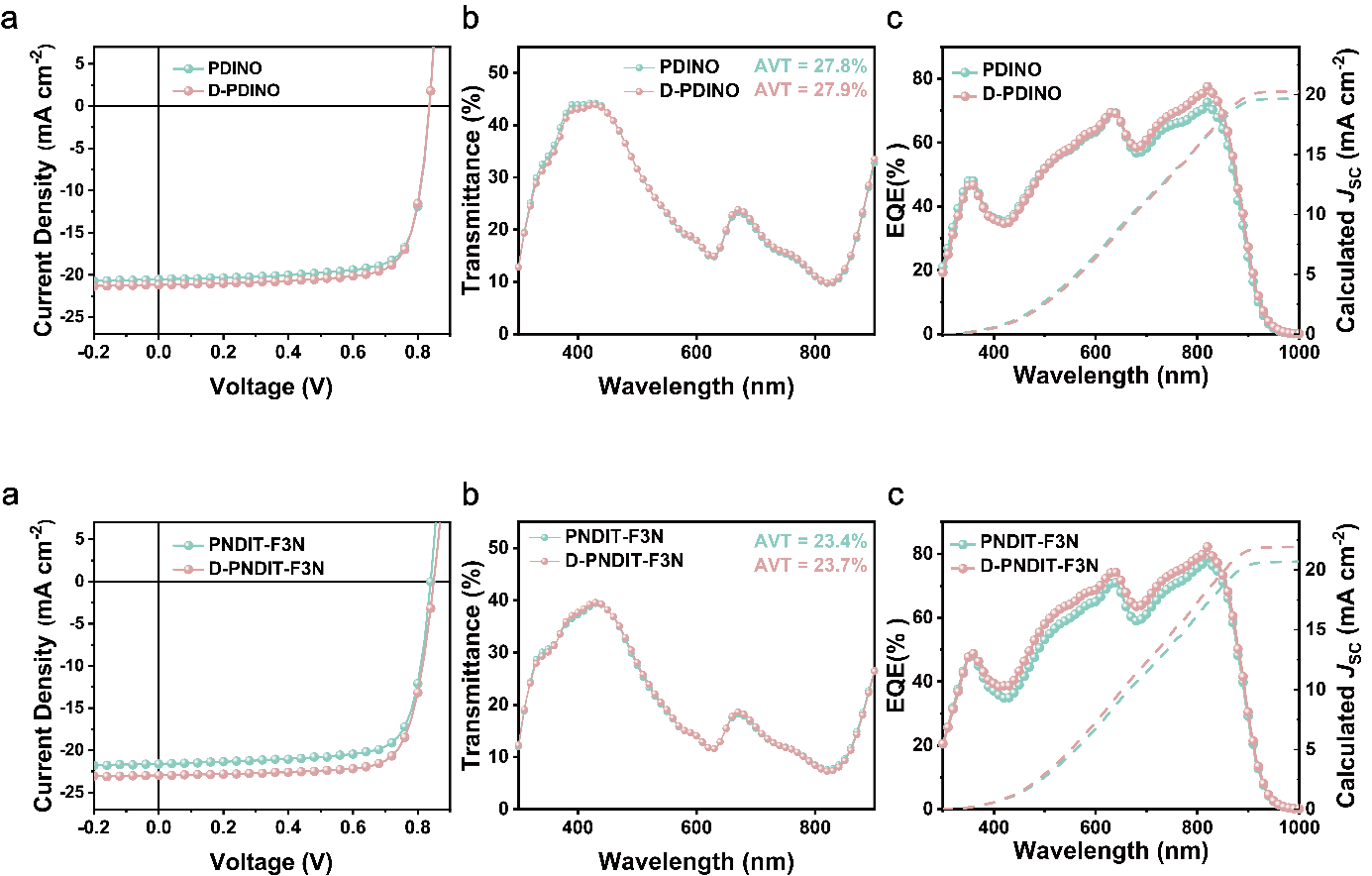


**Fig. S12** (**a**) The *J*–*V* curves, (**b**) the transmittance spectra and (**c**) EQE spectra of STOPVs based on PDINO and D-PDINO


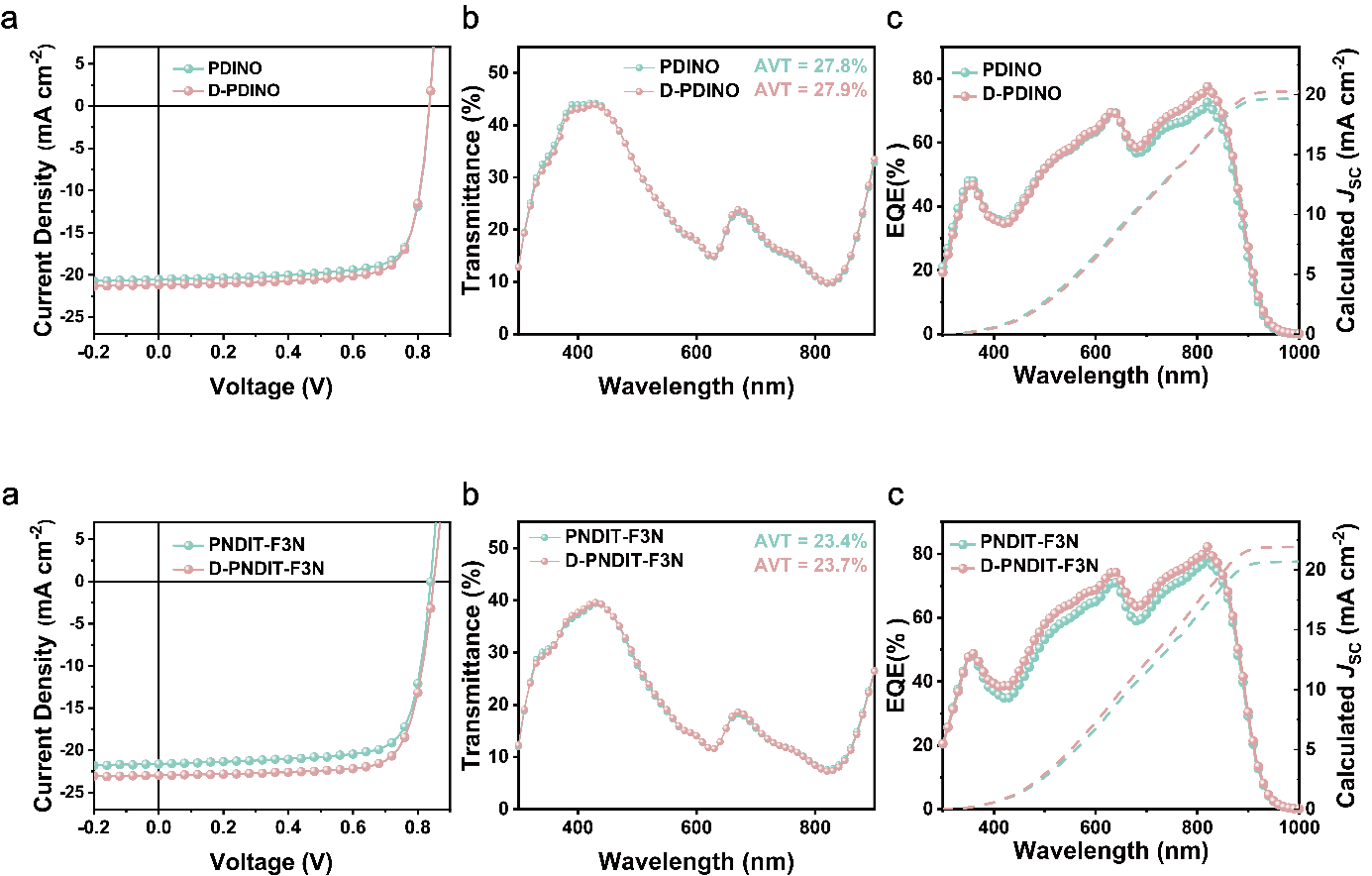


**Fig. S13** (**a**) The *J*–*V* curves, (**b**) the transmittance spectra and (**c**) EQE spectra of STOPVs based on PNDIT-F3N and D-PNDIT-F3N


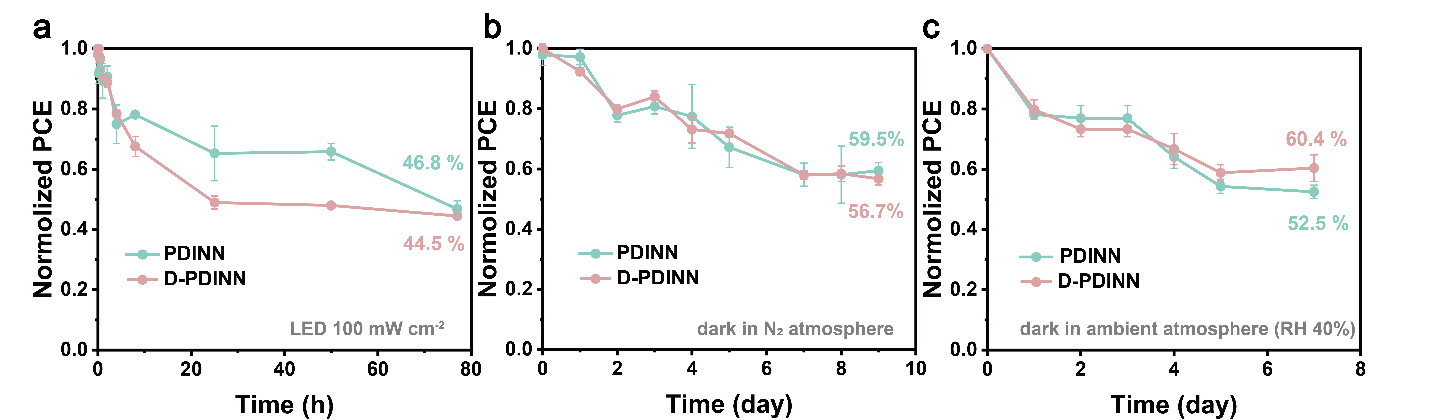


**Fig. S14** The stability of PDINN or D-PDINN based STOPVs (**a**) under light (LED 100 mW cm^–2^), storage in (**b**) N_2_ atmosphere and (**c**) ambient atmosphere (RH 40%) under dark


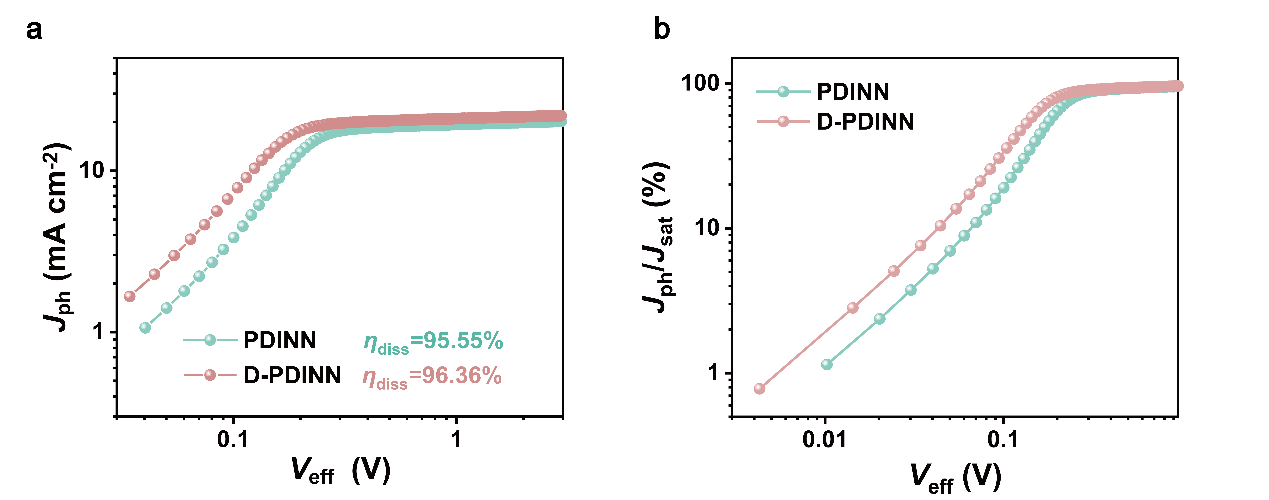


**Fig. S15** (**a**) Photocurrent density (*J*_ph_) at different effective voltage (*V*_eff)_. (**b**) *J*_ph_/*J*_sat_ at different *V*_eff_


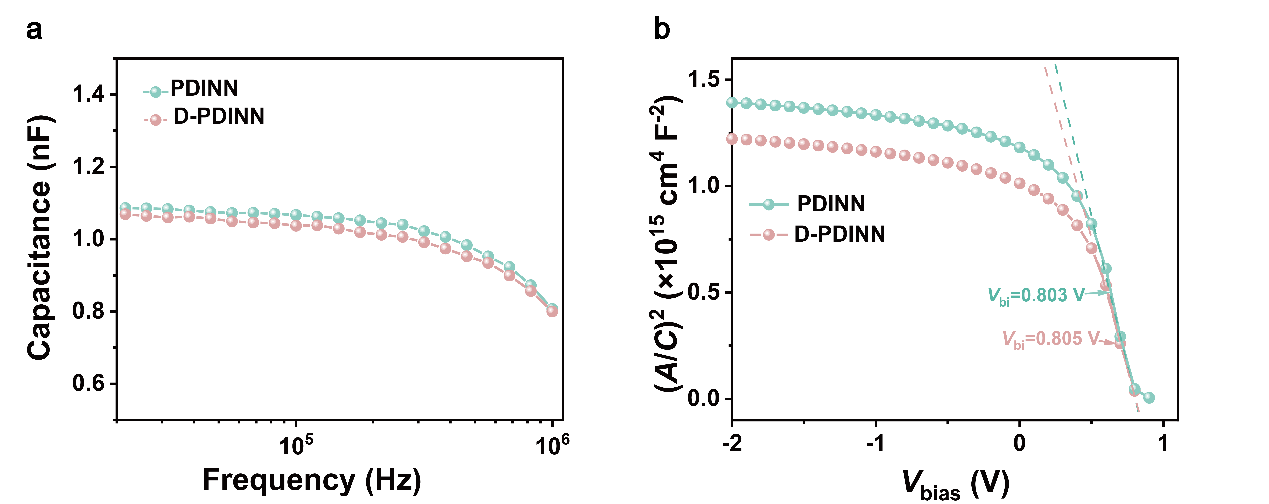


**Fig. S16** (**a**) Capacitance spectra and (**b**) Mott-Schottky curves of in the dark of D-PDINN and PDINN based STOPVs


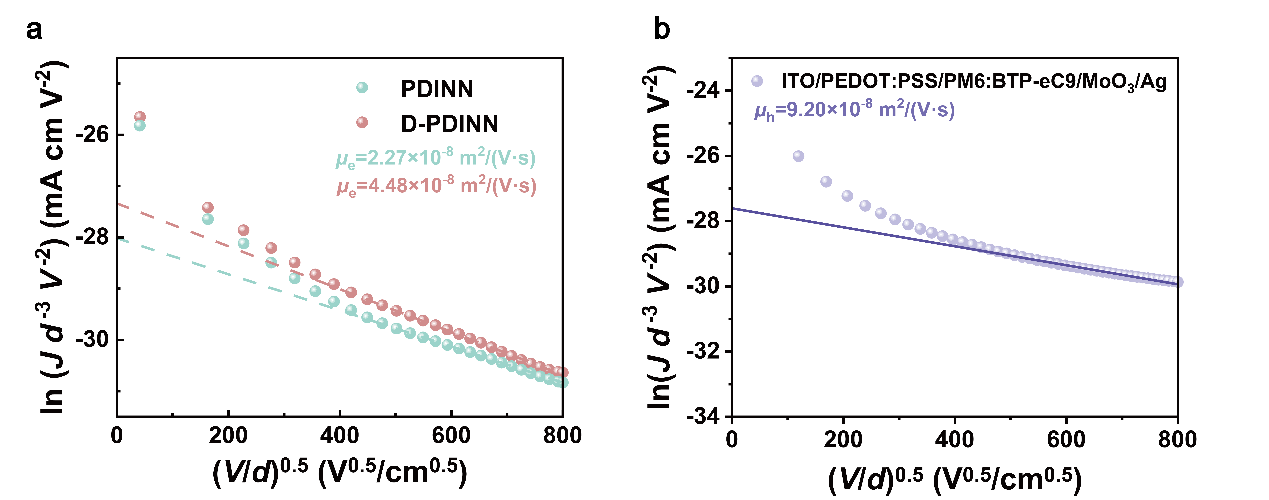


**Fig. S17** (**a**) *J*–*V* characteristics in dark for electron-only devices with different ETLs. (**b**) *J-V* characteristics in dark for hole-only device

**Table S1** Summary of optical structure-free STOPVs reported in literatures

| Device structure | PCE (%) | AVT (%) | LUE (%) | Refs. |
| --- | --- | --- | --- | --- |
| ITO/TBT-L-Br/PM6:BTP-eC9/Ag (15 nm) | 14.57 | 17.11 | 2.49 | [S8] |
| ITO/TBT-L-Br/PM6:L8-BO:BTP-eC9/Cu (10 nm) | 12.26 | 32.94 | 4.04 | [S8] |
| ITO/PEDOT:PSS/PM6:Y6:PC_71_BM/Au (1 nm)/Ag (10 nm) | 11.66 | 24.95 | 2.90 | [S9] |
| ITO/PEDOT:PSS/PM6/BTP-eC9/PDINN/Ag (12 nm) | 11.33 | 19.28 | 2.18 | [S10] |
| ITO/2PACz-SAI/PBDB-TF:L8-BO:BTP-eC9/PDINN/Ag (10 nm) | 13.11 | 28.62 | 3.75 | [S11] |
| ITO/PEDOT:PSS/PM6:Y6:SN/PDINN/Ag (10 nm) | 14.0 | 20.2 | 2.82 | [S12] |
| ITO/PEDOT:PSS/PBDB-TF:Y6:PC_71_BM/PDINO/Ag (15 nm) | 13.0 | 21.4 | 2.78 | [S13] |
| ITO/ZnO/PM6:Y6/MoO_3_/PH1000:AL4083/Au (1 nm)/Ag (15 nm) | 13.7 | 22.2 | 2.97 | [S14] |
| ITO/PEDOT:PSS/D18:N3/PDIN/Au (1 nm)/Ag (10 nm) | 12.91 | 22.49 | 2.90 | [S15] |
| ITO/2PACz/PM6/Y6/PNDIT-F3N-Br/Au (1 nm)/Ag (8 nm) | 11.10 | 30.61 | 3.40 | [S16] |
| ITO/ZnO/PM6:Y6 (*N*-DMBI) /MoO_3_/Ag (20 nm) | 13.03 | 21.9 | 2.86 | [S17] |
| ITO/PEDOT:PSS/PM6:BTP-eC9/ZnO/11-MUA/Ag (12 nm) | 12.1 | 27.0 | 3.27 | [S18] |
| ITO/PEDOT:PSS/PM6:L8-BO/PDINN/Au(1 nm)/Ag (15 nm) | 12.3 | 20.63 | 2.54 | [S19] |
| ITO/PEDOT:PSS/PM6:Y6 (TrTPFB) /PDINO/Ag (11 nm) | 11.1 | 28.7 | 3.18 | [S20] |
| ITO/PEDOT:PSS/PM6:S9SBO-F:Y6-O/PNDIT-F3N/Ag (11 nm) | 12.42 | 24.42 | 3.03 | [S21] |
| ITO/CuSCN/PM6/BTP-eC9/PDINN/Au(2 nm)/Ag (10 nm) | 10.8 | 30.9 | 3.34 | [S22] |
| ITO/PEDOT:PSS/PM6:BTP-eC9:L8-BO/ZnO/Sm:Ag (12 nm) | 10.09 | 32.0 | 3.40 | [S23] |

| ETL | V_OC_ (V) | *J*_SC_  (mA cm^−2^) | FF  (%) | PCE  (%)^a^ | cal. *J*_SC_  (mA cm^−2^)^b^ |
| --- | --- | --- | --- | --- | --- |
| PDINN | 0.844  (0.843±0.001) | 28.7  (29.0±0.4) | 77.0  (76.4±0.9) | 18.7  (18.7±0.1) | 27.3 |
| D-PDINN | 0.846  (0.846±0.001) | 29.2  (29.3±0.2) | 78.3  (77.8±0.5) | 19.4  (19.3±0.1) | 27.8 |

**Table S2** Device data of opaque OPVs with PDINN or D- PDINN

*^a^* Averaged values with standard deviation in parentheses were obtained from 5 devices.

*^b^* *J*_SC_ calculated from the integration of EQE spectra with the AM 1.5G spectrum.

**Table S3** Device data of STOPVs with PDINO, D-PDINO, PNDIT-F3N and D- PNDIT-F3N

| ETL | V_OC_ (V) | *J*_SC_  (mA cm^−2^) | FF  (%) | PCE  (%)^a^ | cal. *J*_SC_  (mA cm^−2^)^b^ | AVT  (%)^c^ | LUE  (%) |
| --- | --- | --- | --- | --- | --- | --- | --- |
| PDINO | 0.833  (0.833±0.001) | 20.5  (20.4±0.2) | 76.6  (76.4±0.5) | 13.1  (13.0±0.1) | 19.7 | 27.8 | 3.64 |
| D-PDINO | 0.833  (0.833±0.003) | 21.3  (21.3±0.2) | 76.5  (76.4±0.4) | 13.7  (13.6±0.1) | 20.3 | 27.9 | 3.82 |
| PNDIT-F3N | 0.838  (0.839±0.003) | 21.6  (21.4±0.2) | 75.6  (75.9±0.7) | 13.7  (13.6±0.1) | 20.7 | 23.4 | 3.21 |
| D-PNDIT-F3N | 0.848  (0.842±0.004) | 23.0  (22.9±0.2) | 76.0  (76.4±0.8) | 14.9  (14.7±0.1) | 21.9 | 23.7 | 3.53 |

*^a^* Averaged values with standard deviation in parentheses were obtained from 5 devices.

*^b^* *J*_SC_ calculated from the integration of EQE spectra with the AM 1.5G spectrum.

*^c^* Arithmetic mean of transmittance in 400–700 nm.

**Supplementary References**

1. F. Neese, The *ORCA* program system. Wires Comput. Mol. Sci. **2**(1), 73–78 (2012). <https://doi.org/10.1002/wcms.81>
2. F. Neese, Software update: The *ORCA* program system: Version 5.0. Wires Comput. Mol. Sci. **12**(5), e1606 (2022). <https://doi.org/10.1002/wcms.1606>
3. C. Lee, W. Yang, R. Parr, Development of the *Colle*-Salvetti correlation-energy formula into a functional of the electron density. Phys. Rev. B **37**(2), 785–789 (1988). <https://doi.org/10.1103/physrevb.37.785>
4. A.D. Becke, Density-functional thermochemistry. III. The role of exact exchange. J. Chem. Phys. **98**(7), 5648–5652 (1993). <https://doi.org/10.1063/1.464913>
5. W. Humphrey, A. Dalke, K. Schulten, VMD: Visual molecular dynamics. J. Mol. Graph. **14**(1), 33–38 (1996). <https://doi.org/10.1016/0263-7855(96)00018-5>
6. F. Weigend, R. Ahlrichs, Balanced basis sets of split valence, triple *Zeta* valence and quadruple *Zeta* valence quality for H to Rn: Design and assessment of accuracy. Phys. Chem. Chem. Phys. **7**(18), 3297–3305 (2005). <https://doi.org/10.1039/B508541A>
7. T. Lu, F. Chen, Multiwfn: a multifunctional wavefunction analyzer. J. Comput. Chem. **33**(5), 580–592 (2012). <https://doi.org/10.1002/jcc.22885>
8. Z. You, J. Wen, W. Liu, Z. Fink, X. Wu et al., Transparent and conductive polyimide-ionene hybrid interlayers for high performance and cost-effective semitransparent organic solar cells. Adv. Mater. **37**(15), e2500450 (2025). <https://doi.org/10.1002/adma.202500450>
9. C.-P. Chen, C.-C. Lee, B.-H. Jiang, M.-W. Hsu, Y.-Y. Yu, Overcoming light loss in semi-transparent ternary organic solar cells with ultra-thin metal electrodes. Org. Electron. **138**, 107174 (2025). <https://doi.org/10.1016/j.orgel.2024.107174>
10. J.Y. Kim, S.J. Jeon, H.S. Lee, Y.W. Han, Y.C. Kim et al., Distribution of dual additives enables efficient semi-transparent layer-by-layer architecture of organic solar cells. J. Mater. Chem. C **12**(16), 5908–5915 (2024). <https://doi.org/10.1039/D3TC02932H>
11. S. Guan, Y. Li, C. Xu, N. Yin, C. Xu et al., Self-assembled interlayer enables high-performance organic photovoltaics with power conversion efficiency exceeding 20%. Adv. Mater. **36**(25), 2400342 (2024). <https://doi.org/10.1002/adma.202400342>
12. W. Liu, S. Sun, L. Zhou, Y. Cui, W. Zhang et al., Design of near-infrared nonfullerene acceptor with ultralow nonradiative voltage loss for high-performance semitransparent ternary organic solar cells. Angew. Chem. Int. Ed. **61**(19), e202116111 (2022). <https://doi.org/10.1002/anie.202116111>
13. N. Zhang, T. Jiang, C. Guo, L. Qiao, Q. Ji et al., High-performance semitransparent polymer solar cells floating on water: Rational analysis of power generation, water evaporation and algal growth. Nano Energy **77**, 105111 (2020). <https://doi.org/10.1016/j.nanoen.2020.105111>
14. Y. Zhao, P. Cheng, H. Yang, M. Wang, D. Meng et al., Towards high-performance semitransparent organic photovoltaics: dual-functional p-type soft interlayer. ACS Nano **16**(1), 1231–1238 (2022). <https://doi.org/10.1021/acsnano.1c09018>
15. C. Xu, K. Jin, Z. Xiao, Z. Zhao, X. Ma et al., Wide bandgap polymer with narrow photon harvesting in visible light range enables efficient semitransparent organic photovoltaics. Adv. Funct. Mater. **31**(52), 2107934 (2021). <https://doi.org/10.1002/adfm.202107934>
16. H. Zhang, L. Xiao, Y. Zhang, S. Li, W. Zhang et al., Fine-tuning thickness-dependent molecular aggregation for enhanced performance in semitransparent organic photovoltaics. Sol. RRL **9**(2), 2400745 (2025). <https://doi.org/10.1002/solr.202400745>
17. J. Xie, W. Lin, K. Zheng, Z. Liang, N-doping donor-dilute semitransparent organic solar cells to weaken donor: acceptor miscibility and consolidate donor-phase continuity. Adv. Sci. **11**(31), 2404135 (2024). <https://doi.org/10.1002/advs.202404135>
18. H. Yu, J. Wang, Y. Li, T. Liu, Y. Gong et al., Mitigating interfacial recombination enabling efficient semitransparent organic photovoltaics. Chem. Eng. J. **499**, 156475 (2024). <https://doi.org/10.1016/j.cej.2024.156475>
19. D. Wang, F. Zhang, M. Li, Y. Liu, A. Geng et al., Optimization light utilization efficiency of semitransparent organic solar cells by regulating absorption spectrum. ACS Appl. Energy Mater. **7**(14), 6013–6020 (2024). <https://doi.org/10.1021/acsaem.4c01498>
20. F. Xue, Y. Xie, Y. Cui, D. Yu Paraschuk, W. Ma et al., Boosting fill factor of semitransparent donor-poor organic solar cells for the best light utilization efficiency. Adv. Funct. Mater. **35**(8), 2415617 (2025). <https://doi.org/10.1002/adfm.202415617>
21. N. Zhang, Z. Zhou, Y. An, F. Qi, R. Xia et al., Modeling-guided design of semitransparent organic photovoltaics with improved energy harvesting and saving capabilities. Adv. Energy Mater. **16**(3), 2404129 (2026). <https://doi.org/10.1002/aenm.202404129>
22. X. Yan, J. Wang, W. He, T.A. Dela Peña, C. Zhu et al., Semitransparent organic photovoltaics enabled by transparent p-type inorganic semiconductor and near-infrared acceptor. J. Energy Chem. **96**, 351–358 (2024). <https://doi.org/10.1016/j.jechem.2024.05.008>
23. M. Zhao, J. Wu, H. Tang, X. Yi, Z. Liu et al., A novel ultra-thin and smooth Sm: Ag composite electrode with high visible transmittance enables efficient color-neutral semitransparent organic solar cells. Adv. Opt. Mater. **12**(35), 2401776 (2024). <https://doi.org/10.1002/adom.202401776>
